# A novel lncRNA, Lnc21q22.11, suppresses gastric cancer growth by inhibiting MEK/ERK pathway

**DOI:** 10.1080/15592294.2025.2512764

**Published:** 2025-06-02

**Authors:** Cheng Zhu, Meiying Zhang, Weili Yang, Aiai Gao, Xiaoyuan Yu, Xiaomo Su, Runsheng Chen, Mingzhou Guo

**Affiliations:** a School of Medicine, NanKai University, Tianjin, China; b Department of Gastroenterology and Hepatology, The First Medical Center, Chinese PLA General Hospital, Beijing, China; c Key Laboratory of RNA Biology, Institute of Biophysics, Chinese Academy of Sciences, Beijing, China; d National Key Laboratory of Kidney Diseases, The First Medical Center, Chinese PLA General Hospital, Beijing, China

**Keywords:** Gastric cancer, Lnc21q22.11, long non-coding RNA, targeting therapy, MEK/ERK signaling pathway

## Abstract

Gastric cancer (GC) is one of the most common malignancies with limited treatment options and poor prognosis. Therefore, it is necessary to identify new markers for the development of novel therapeutic strategies. Long non-coding RNAs (lncRNAs) have emerged as pivotal players in cancer. However, RNA-based cancer therapy has been challenged by non-specificity and adverse immune effects. Thus, a comprehensive understanding of the functional roles of lncRNAs and their regulatory networks in downstream pathways may provide more specific targets. In this study, we identified a novel lncRNA, Lnc21q22.11, encoded by the region of chromosome 21q22.11. The full-length transcript was 1202 nt, and its expression was reduced in GC. The expression of Lnc21q22.11 was regulated by histone methylation. Lnc21q22.11 inhibited GC cell proliferation, colony formation, invasion, and migration. Lnc21q22.11 suppressed N87 cell xenograft growth in mice. Mechanistically, Lnc21q22.11 inhibited the mitogen-activated protein kinase kinase/extracellular signal-regulated kinase (MEK/ERK) signaling pathway by interacting with MYH9 in GC cells. Loss of or reduced Lnc21q22.11 expression sensitized GC cells to MEK inhibitor. In conclusion, Lnc21q22.11 is a novel lncRNA in gastric cancer. It suppresses gastric cancer growth by inhibiting the MEK/ERK signaling pathway both *in vitro* and *in vivo*.

## Key policy highlights

• Lnc21q22.11 is a novel lncRNA with the full length obtained.

• The expression of Lnc21q22.11 is regulated by histone modification.

• Lnc21q22.11 suppresses gastric cancer growth both *in vitro* and *in vivo*.

• Lnc21q22.11 inhibits MEK/ERK signaling pathway by interacting with MYH9.

## GRAPHICAL ABSTRACT

**Figure:**
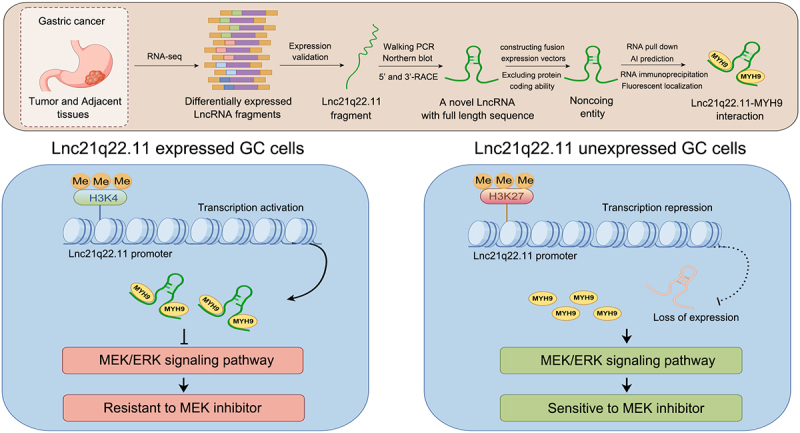
Working model for the isolation, identification of Lnc21q22.11, and mechanical study

Me: methylation

## Introduction

The incidence of gastric cancer (GC) is geographically distinct worldwide, with a high prevalence in Asian countries [1]. Approximately 10% of gastric cancer patients show familial aggregation, and 1–3% of cases have germline mutations, including APC, MLH1, MLH2, PMS2, MSH6, PTEN, SMAD4, MUTYH, STK11, and other genes [2,3]. However, genetic alterations remain unknown in about 70% of hereditary gastric cancer patients. Modifiable risk factors include Helicobacter pylori and Epstein-Barr virus infections, diet, smoking, alcohol consumption, and environmental factors [4,5]. The interactions of these factors promote GC development. Despite advances in cancer biology and treatment, prognosis remains poor, and the median overall survival is about 1 year for advanced-stage GC [6]. For precision medicine, HER2 positivity, PD-L1 expression, and microsatellite instability-high status are the only effective markers of targeted therapies [6]. Therefore, it is necessary to deeply understand the mechanisms of GC and innovate therapeutic strategies.

RNA biology has revolutionized the understanding and treatment of cancer. Dysregulation of non-coding RNAs (ncRNAs) influences gene regulatory networks and contributes to the hallmarks of cancer [7], among which long non-coding RNAs (lncRNAs) were discovered long before the genomic era. However, their extensive biological functions have not been well recognized to date [8–10]. Although many lncRNAs have been reported, only a handful of lncRNAs with full-length sequences have been obtained and functionally characterized [10–12]. Recent findings indicate that lncRNAs play a determining role in cell phenotype and fate [13,14]. RNA-based cancer therapy remains challenging due to off-target effects caused by sequence similarities [15]. The identification of novel cancer-related lncRNAs and the clarification of their regulatory signaling pathways may lead to the development of more specific and effective therapeutic approaches.

Herein, we identified a novel lncRNA which was 1202 nt in length. It was located on chromosome 21q22.11 and designated as Lnc21q22.11. Reduced expression of Lnc21q22.11 was detected in GC, and its expression was regulated by histone modifications. Lnc21q22.11 inhibited the mitogen-activated protein kinase kinase/extracellular signal-regulated kinase (MEK/ERK) signaling pathway by interacting with MYH9. Loss of/reduced Lnc21q22.11 expression sensitized GC cells to MEK inhibitor.

## Materials and methods

### Primary cancer samples and cell lines

Matched cancerous and noncancerous tissue samples were obtained from the Chinese PLA General Hospital. The study was performed in accordance with the Declaration of Helsinki and was approved by the Institutional Review Board of the Chinese PLA General Hospital (IRB number: 20090701–015). Informed written consent was obtained from all individuals included in this study. GC cell lines were cultured in RPMI-1640 medium or Dulbecco’s modified Eagle’s medium (Gibco, #11875093, #11965092) supplemented with 10% fetal bovine serum (Gibco, #10099141C) and penicillin-streptomycin (MCE, #HY-K1006), including AGS, N87, BGC-823, MGC-803, GES-1, PHM82, NUGC-3, SNU-1, SNU-5, SNU-16, SGC-7901, and MKN45 cells.

### DNA extraction and modification, RNA preparation, RT-PCR and methylation-specific PCR

DNA and RNA preparation, as well as sodium bisulfite modification, were performed as previously described [16]. The sequences of RT-PCR and methylation-specific PCR primers are listed in Supplementary table S1.

### Northern blotting, 5'- and 3'- rapid amplification of cDNA ends

Biotin-labeled Lnc21q22.11 probes were synthesized with T7 RNA polymerase using a biotin RNA labeling mix (NEB, USA, #M0251). The sequences of the labeling probes are listed in Supplementary table S1, and the probes were purified using RNA Clean & Concentrator-5 kit (ZYMO Research, USA, #R1013). Total RNA from BGC-823 cells was separated on 0.8% denaturing formaldehyde agarose gels in MOPS running buffer (Thermo, USA, #AM8671) and transferred to nylon-membrane-N+ (Ambion, USA, #AM10104) in SSC buffer (Thermo, #AM9763) overnight. Northern blotting was performed using a Chemiluminescent Nucleic Acid Detection Module (Thermo, #89880) according to the manufacturer’s instructions after ultraviolet crosslinking.

The 5'- and 3'- rapid amplification of cDNA ends (5'-RACE and 3'-RACE) were performed following the manufacturer’s instructions for the FirstChoice^TM^ RLM-RACE kit (Ambion, #AM1700M). The primer sequences are listed in Supplementary table S1.

### RNA fluorescence in situ hybridization

RNA fluorescence in situ hybridization (FISH) was performed to detect the subcellular localization of Lnc21q22.11 in viable cells, following the manufacturer’s protocol (Beyotime, China, #R0306). Briefly, cells were washed with PBS, fixed with 4% formaldehyde, permeabilized with PBS containing 0.5% Triton X-100 (Sigma, USA, #X100), and washed three times with PBS. Labeled probes were added following the instructions of the Cy3 TSA fluorescence system (APExBIO, USA, #K1051). The subcellular localization of Lnc21q22.11 was observed using a Leica TCS SP2 confocal laser microscope.

### Establishment of Lnc21q22.11 stable or transient expression GC cells and validation of Lnc21q22.11 as a non-protein coding transcript

Full-length Lnc21q22.11 cDNA was obtained by RT-PCR amplification and cloned into the pLenti6-GFP expression vectors, and the primer sequences are listed in Supplementary table S1. After transfection into HEK-293T cells using Lipofectamine 3000 reagent (Invitrogen, USA, #L3000001), the supernatant containing the enveloped virus was collected. After infecting AGS and N87 cells, Lnc21q22.11 stably-expressed cells were obtained through blasticidin selection and limited dilution assays. Transient Lnc21q22.11 expression vector was constructed using the pcDNA3.1 plasmid.

Supposing three potential start codons exist in the 5' of Lnc21q22.11, we constructed three potential coding start sites of Lnc21q22.11 constructs, including 5'-ATG-*GCTCAAC* (Lnc21q22.11 sequence)-Flag-3,' 5'-ATG-*CTCAAC* (Lnc21q22.11 sequence)-T-Flag-3,' and 5'-ATG-*TCAAC* (Lnc21q22.11 sequence)-TT-Flag-3.' The ZSCAN23-Flag construct was used as a protein expression control [17].

### Methyl thiazolyl tetrazolium, flow cytometry, transwell assays

The methyl thiazolyl tetrazolium (MTT) assay was used to evaluate cell viability. Cells were seeded in 96-well plates at a density of 2 × 10^3^ cells/well and cultured for 0, 24, 48, 72, and 96 h (KeyGEN Biotech, China, #KGT5251). Each experiment was repeated three times and the absorbance was measured by microplate reader (Thermo Multiskan MK3, USA).

Cell cycle and apoptosis were analysed by flow cytometry. After growth for 12 h in FBS-free RPMI 1640 medium for synchronization, the complete medium was changed to continuous culture for 24 h. GC cells were then fixed with 70% ethanol and stained with iodide (KeyGEN Biotech, #KGA9101) for FACS Caliber flow cytometry detection (BD Biosciences, USA). ModFit software was used for cell phase analysis (Verity Software House). Cell apoptosis was examined using the Annexin V-FITC/PI Apoptosis Detection Kit, according to the manufacturer’s instructions (KeyGEN Biotech, #KGA1101). Each experiment was repeated three times.

Cell migration and invasion were examined using a transwell assay. To evaluate cell migration ability, AGS, N87, and BGC-823 cells (1 × 10^5^ cells in 200 µL volume) were added to the upper chamber of the transwell apparatus (Corning, USA, #3412) and incubated for 30, 30, and 36 h, respectively. For invasion detection, a layer of extracellular matrix was coated in the upper chamber (Corning, #354234). These cells were grown for 36, 36, and 48 h, respectively. The cells were fixed with 75% ethanol and stained with crystal violet for counting. The experiment was repeated three times.

### GC cell xenograft mouse model

Four-week-old BALB/c nude mice were purchased from SPF Company (Beijing, China), and were randomly divided into 2 groups (7 mice per group). N87 cells without expression and re-expression of Lnc21q22.11 were inoculated subcutaneously into the mice, respectively. Tumour volume was measured every four days starting from the fourth day after inoculation for a total of 20 days. After measurement on day 20, the mice were euthanized by cervical dislocation in strict adherence to the American Veterinary Medical Association (AVMA) Guidelines (2020). The animal experiment was performed following ARRIVE guidelines and approved by the Animal Ethics Committee of the Chinese PLA General Hospital (approval number: 2022-X18-77) (https://arriveguidelines.org/sites/arrive/files/documents/Author%20Checklist%20-%20E10%20only.pdf).

### Chromatin immunoprecipitation

Chromatin immunoprecipitation (ChIP) PCR was performed following the instructions of the EpiTect ChIP One Day Kit (Qiagen, Germany, #334471). H3K4me3, H3K9me2, H3K27me3, and IgG antibodies (Abclonal, China, #A22226, #A2359, #A22006, Proteintech, China, #10284) were used for the ChIP assay. The ChIP-PCR primer sequences are listed in Supplementary table S1.

### Western blotting, RNA pull-down assay, and RNA immunoprecipitation

Western blotting was performed as previously described [17]. The following antibodies were used: CyclinA2 (Proteintech, #18202), CylinD1 (Proteintech, #60186), CyclinE1 (Proteintech, #11554), Bax (Proteintech, #50599), Bcl2 (Proteintech, #68103), Caspase3/Cleaved-caspase3 (Proteintech, #19677), MMP2 (Proteintech, #10373), MMP7 (Proteintech, #10374), MMP9 (Proteintech, #10375), β-actin (Proteintech, #66009), MEK (Proteintech, #11049), p-MEK (Proteintech, #28930), ERK1/2 (Proteintech, #66192), p-ERK1/2 (Proteintech, #28733), c-myc (Proteintech, #10828), P38 (CST, USA, #9212), p-P38 (CST, #9211), JNK (Proteintech, #17572), p-JNK (Proteintech, #80024).

RNA pull-down assay was performed using biotin-labeled Lnc21q22.11 probes/LacZ probes, and streptavidin beads, following the the manufacturer’s protocol (Invitrogen, #65602). The sequences of the probes are listed in Supplementary table S1.

RNA immunoprecipitation (RIP) was performed using MYH9 and rabbit IgG antibodies (Proteintech, #11128, #10284) in Lnc21q22.11 highly expressed BGC-823 cells, following the manufacturer’s protocol (Thermo, #26162).

### Statistical analysis

The χ2 test and two-tailed Student’s *t*-test were performed for statistical analysis using the SPSS software (version 18.0, USA) and GraphPad Prism 8.0 software (GraphPad Software Inc., USA), respectively. *p* < 0.05 was considered statistically significant.

## Results

### The levels of Lnc21q22.11 were decreased in GC tissue samples

**Figure 1.**
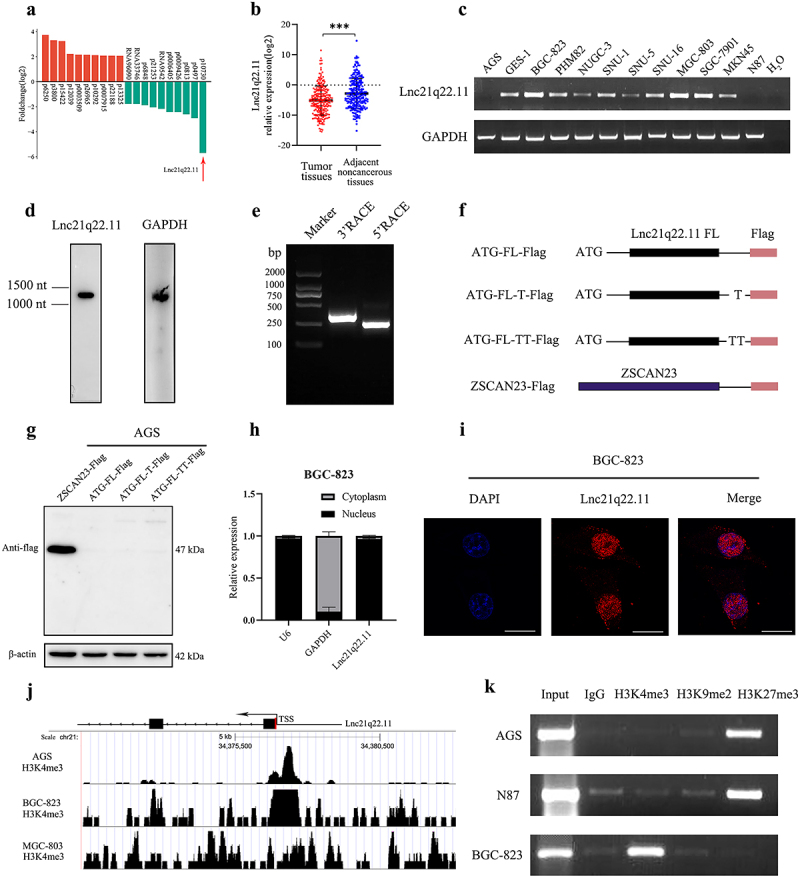
Isolation and identification of Lnc21q22.11 in gastric cancer. (a) RNA-seq data showing the Fold changes in lncRNA expression levels in gastric cancer compared to adjacent tissue samples. (b) Real-time PCR results showing reduced expression of Lnc21q22.11 in cancer tissue compared to adjacent tissue samples (*n* = 245). ***p < 0.001. (c) RT-PCR results showing the expression of Lnc21q22.11 in gastric cancer cell lines. H_2_O: negative control; GAPDH: internal control. (d) Northern blotting showing the size of Lnc21q22.11, 1000 nt and 1500 nt were marker sizes. GAPDH served as an RNA expressing control. (e) 5'-RACE and 3'-RACE results obtained after walking-PCR extension. (f) Three potential coding patterns of the full length Lnc21q22.11 sequence were constructed into pcDNA3.1 expression vectors. ZSCAN23-Flag fusion protein served as a positive control. FL: full length sequence. (g) Different patterns of Lnc21q22.11 constructs were transfected into AGS cells and detected with Flag antibody. The size of ZSCAN23-Flag was 47 kDa. β-actin: internal control. (h) Cytoplasmic and nuclear RNA were separated from BGC-823 cells, and Lnc21q22.11 was detected by real-time PCR. U6: control for nucleus; GAPDH: control for cytoplasm. (i) FISH assay showing localization of Lnc21q22.11 in BGC-823 cells. The scale bar represents 25 μm. (j) Histone modification status of the Lnc21q22.11 promoter region predicted by cistrome DB database. H3K4me3 enrichment locations indicate transcriptional activation sites in AGS, BGC-823, and MGC-803 cells. (k) Histone modification status in Lnc21q22.11 promoter region detected by ChIP-PCR assay with H3K4me3, H3K9me2, H3K27me3 antibodies.

The fragment of Lnc21q22.11 transcript was discovered by analysing four types of cancer and matched noncancerous tissue samples using RNA-seq (GSA for human, accession number: HRA011214). The original transcript length was 471 nt, with the most significant expression discrepancy observed between cancer and adjacent tissue samples (Figure 1(a)). The differential expression of Lnc21q22.11 was further validated by real-time PCR in 245 pairs of GC and adjacent noncancerous tissue samples. The expression level of Lnc21q22.11 was significantly lower in GC tissues than in adjacent noncancerous tissues (*p* < 0.001, Figure 1(b)). Reduced expression of Lnc21q22.11 was found in 73.5% (180/245) of GC tissue samples (Table 1). Its expression was associated with tumour size (*p* < 0.05, Table 1), and no association was observed with other factors, including gender, age, TNM stage, tumour differentiation, lymph node metastasis, smoking or drinking status, or family history (all *p* > 0.05, Table 1).

**Table 1.** Association between Lnc21q22.11 expression and clinical-pathological characteristics of GC patients.

Features		n	Low	High	χ^2^	*P* value
All cases		245	180 (73.5%)	65 (26.5%)		
Gender						
	Female	68	53	15	0.966	0.326
	Male	177	127	50		
Age (year)						
	<60	88	60	28	1.970	0.160
	≥60	157	120	37		
Smoking status						
	Yes	84	59	25	0.685	0.408
	No	161	121	40		
Drinking status						
	Yes	58	41	17	0.301	0.583
	No	187	139	48		
Family history						
	Yes	81	59	22	0.025	0.875
	No	164	121	43		
Tumor size (cm)						
	≤6	184	129	55	4.282	0.039*
	>6	61	51	10		
Differentiation						
	High/Moderate	56	42	14	0.087	0.768
	Low	189	138	51		
Lymph node metastasis						
	Yes	183	138	45	1.397	0.237
	No	62	42	20		
TNM stage						
	I<II	104	73	31	0.996	0.318
	III<IV	141	107	34		

*p* values were obtained from χ^2^ test, *p < 0.05.

### The full-length of Lnc21q22.11 is 1202 nt

To further investigate the function of Lnc21q22.11 fragment, its expression was detected in 12 GC cell lines. High levels of expression were observed in BGC-823 and MGC-803 cells, moderate expression was observed in GES-1, PHM82, NUGC-3, SNU-1, SNU-5, SNU-16, SGC-7901, and MKN45 cells, and loss of expression was found in AGS and N87 cells (Figure 1(c)).

Then the full-length of Lnc21q22.11 was evaluated by northern blotting using labeled probes derived from the original fragment. As shown in Figure 1(d), the band was located between 1000 nt and 1500 nt. By referencing the genomic sequences, we developed a walking PCR assay. Combining 3'-RACE and 5'- RACE with walking PCR assays, the full-length sequence of Lnc21q22.11 was obtained and the full-length is 1202 nt (Figure 1(e), Supplementary table S1). As shown in Supplementary figure S1a, the genomic structure of Lnc21q22.11 comprises 2 exons (556 bp and 646 bp in length, respectively) and 1 intron (4074 bp in length), with the location of RT-PCR primers for Lnc21q22.11 indicated. Partial sequence overlaps were observed between Lnc21q22.11 and the SMIM11, KCNE2, and LOC105372791 (NR_188572.1) genes. To investigate potential regulatory relationships between Lnc21q22.11 and its neighbouring genes, the correlations of their expression levels were analysed in GC cells. No significant correlations were found between Lnc21q22.11 expression and that of SMIM11, KCNE2, or LOC105372791, suggesting that no regulatory roles of Lnc21q22.11 in these adjacent genes (Supplementary figure S1b).

### Lnc21q22.11 is a novel non-protein coding RNA and is mainly localized in the nucleus

To verify Lnc21q22.11 is a non-protein coding RNA, the full-length sequence was cloned into the pcDNA3.1 expression vector by adding ATG to its N-terminal and Flag peptide tag to its C-terminal (Figure 1(f)). Compared to the coding gene control ZSCAN23, no protein bands were observed in the transfected AGS cells, indicating that Lnc21q22.11 lacks protein-coding ability (Figure 1(g)).

Thereafter, the potential open reading frame (ORFs) of Lnc21q22.11 were predicted by ORF Finder (https://www.ncbi.nlm.nih.gov/orffinder/). Two possible peptide-encoding sequences of Lnc21q22.11 were found (Supplementary figure S2a). ORF-mutGFP expression vectors were constructed for the predicted coding peptides and transfected into AGS cells (Supplementary figure S2b,c). No expected protein bands were observed, demonstrating that Lnc21q22.11 has no protein/peptide coding ability (Supplementary figure S2d).

The subcellular localization of Lnc21q22.11 was determined by nuclear-cytoplasmic fractionation and FISH assays in gastric cancer cells. Lnc21q22.11 was predominantly localized in the nucleus of BGC-823 cells (Figure 1(h,i)).

### The expression of Lnc21q22.11 is modulated by histone modification

The regulatory mechanisms of Lnc21q22.11 expression were further explored. First, DNA methylation was excluded as a regulatory factor by analysing the correlation between its expression and methylation status around the transcription start site (Supplementary figure S3). Then data from ChIP-seq database (http://cistrome.org/db/) revealed enriched H3K4me3 modification in the genomic region of the transcription start site in Lnc21q22.11 highly expressed MGC-803 and BGC-823 cells. However, no significant H3K4me3 marker emerged at the same location in Lnc21q22.11 unexpressed AGS cells (Figure 1(j)). Therefore, histone modification markers of transcriptional activation and inhibition were examined by ChIP-PCR in the promoter region of Lnc21q22.11. As shown in Figure 1(k), H3K4me3 was detected in BGC-823 cells and H3K27me3 was observed in AGS and N87 cells, validating the regulatory role of histone modification in Lnc21q22.11 expression.

### Lnc21q22.11 restrains GC cell growth

**Figure 2.**
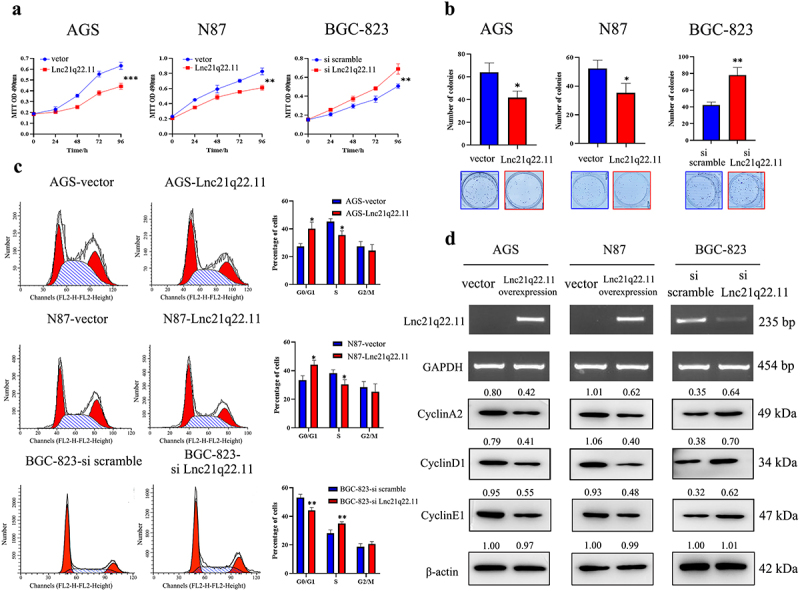
Lnc21q22.11 curbs cell growth and induces cell cycle arrest in GC. (a) Effect of Lnc21q22.11 on cell proliferation detected by MTT assay. (b) Colony formation assay showing that Lnc21q22.11 inhibits GC cell proliferation. (c) Influence of Lnc21q22.11 on the cell cycle in AGS, N87 and BGC-823 cells after re-expression or siRNA knockdown. *p < 0.05, **p < 0.01, ***p < 0.001. (d) Levels of Lnc21q22.11 and cell cycle-related proteins detected by RT-PCR and western blotting. CyclinA2, CyclinD1, CyclinE1 are G1/S phase checkpoint proteins. GAPDH: internal control for RT-PCR; β-acin: internal control for western blotting.

Stable Lnc21q22.11-expressing AGS and N87 cells were established through lentiviral infection, while siRNA was utilized to knock down Lnc21q22.11 expression in BGC-823 cells for biological function analysis. Detected by MTT assay, the optical density (OD) values were 0.63 ± 0.03 *vs*. 0.44 ± 0.02 (*p* < 0.001) and 0.83 ± 0.05 *vs*. 0.61 ± 0.03 (*p* < 0.01) in Lnc21q22.11 unexpressed compared to overexpressed AGS and N87 cells, respectively (Figure 2(a)). For Lnc21q22.11 highly expressed BGC-823 cells, the OD values were 0.51 ± 0.02 vs. 0.69 ± 0.05 (*p* < 0.01) before and after knockdown of Lnc21q22.11 (Figure 2(a)). These results suggest that Lnc21q22.11 inhibits GC cell proliferation.

Colony formation assay was performed to further evaluate the influence of Lnc21q22.11 on cell growth. The number of colonies was 64.00 ± 8.29 vs. 41.67 ± 5.79 (*p* < 0.05) and 52.33 ± 5.73 vs. 35.33 ± 6.18 (*p* < 0.05) in Lnc21q22.11 unexpressed and re-expressed AGS and N87 cells, respectively (Figure 2(b)). The clone number was 42.33 ± 3.68 vs. 78.00 ± 9.18 (*p* < 0.01) in BGC-823 cells before and after knockdown of Lnc21q22.11, supporting the inhibitory role of Lnc21q22.11 in GC cell colony formation (Figure 2(b)).

### Lnc21q22.11 induces G1/S arrest and cell apoptosis in GC cells

The influence of Lnc21q22.11 on the cell cycle and apoptosis was measured using flow cytometry. Before and after restoration of Lnc21q22.11 expression, the percentage of cells in G0/G1 phase was 27.33 ± 2.10% *vs*. 40.12 ± 4.72% (*p* < 0.05) and 33.35 ± 3.13% *vs*. 44.24 ± 3.02% (*p* < 0.05), S phase was 45.17 ± 2.21% *vs*. 35.54 ± 3.10% (*p* < 0.05) and 38.28 ± 2.41% *vs*. 30.53 ± 3.25% (*p* < 0.05), G2/M phase was 27.50 ± 3.43% *vs*. 24.34 ± 4.42% (*p* > 0.05) and 28.37 ± 4.10% *vs*. 25.23 ± 5.54% (*p* > 0.05) in AGS and N87 cells, respectively (Figure 2(c)). In Lnc21q22.11 highly expressed BGC-823 cells, G0/G1 phase was 53.17 ± 2.21% *vs*. 44.21 ± 2.13% (*p* < 0.01), S phase was 28.16 ± 2.35% *vs*. 35.10 ± 1.12% (*p* < 0.01), G2/M phase was 18.67 ± 2.30% *vs*. 20.69 ± 1.78% (*p* > 0.05) before and after knockdown of Lnc21q22.11 (Figure 2(c)). These data imply that Lnc21q22.11 induces G1/S arrest. The effect of Lnc21q22.11 on the cell cycle was further validated by detecting cell cycle-related proteins. Decreased levels of cyclinA2, cyclinD1 and cyclinE1 by Lnc21q22.11 were observed in AGS, N87, and BGC-823 cells, respectively (Figure 2(d)).

**Figure 3.**
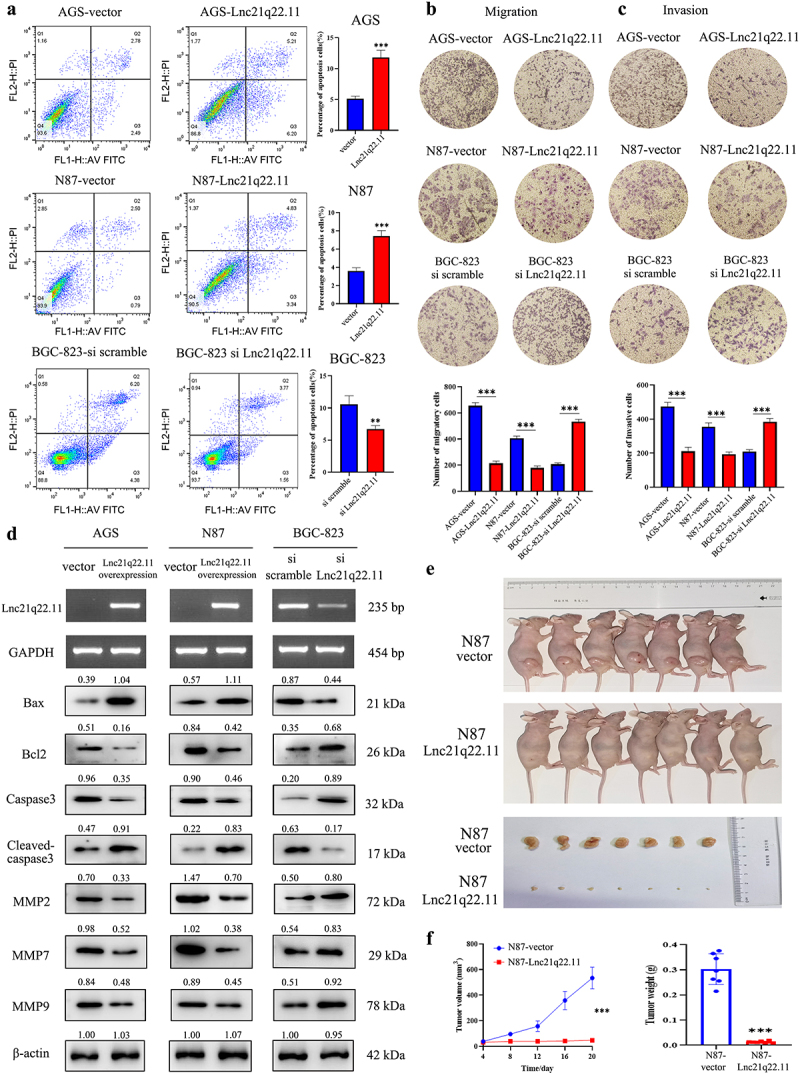
Lnc21q22.11 induces GC cell apoptosis, inhibits cell migration and invasion, and curbs xenografts growth in mice. (a) Expression of Lnc21q22.11 induces cell apoptosis in AGS, N87, and BGC-823 cells. (b)-(c) Representative results of cell migration and invasion assay with or without Lnc21q22.11 expression. (d) Expression of Lnc21q22.11 reduces the levels of Bax, Bcl2, Caspase3, Cleaved-caspase3, MMP2, MMP7 and MMP9 in AGS, N87, and BGC-823 cells. GAPDH: internal control for RT-PCR; β-acin: internal control for western blotting. (e) Lnc21q22.1121q22.11 suppresses N87 cell xenografts growth in mice. (f) Xenograft tumor growth curves and tumor weights in Lnc21q22.11 unexpressed and re-expressed N87 cells. *p < 0.05, **p < 0.01, ***p < 0.001.

The percentage of apoptotic cells was 5.13 ± 0.42% *vs*. 11.81 ± 1.18% (*p* < 0.001) and 3.61 ± 0.35% *vs*. 7.44 ± 0.60% (*p* < 0.001) in AGS and N87 cells, before and after restoration of Lnc21q22.11 expression, respectively (Figure 3(a)). In Lnc21q22.11 highly expressed BGC-823 cells, the percentage of apoptotic cells was 10.57 ± 1.33% *vs*. 6.74 ± 0.51% (*p* < 0.01) before and after knocking down Lnc21q22.11 (Figure 3(a)). These results indicate that Lnc21q22.11 promotes cell apoptosis. Apoptosis-related proteins were examined by western blotting. Reduced levels of Bcl2 and Caspase3, and increased levels of Bax and Cleaved-caspase3 by Lnc21q22.11 were observed in GC cells, further supporting the above results (Figure 3(d)).

### Lnc21q22.11 inhibits GC cells migration and invasion

Transwell assay was used to examine cell migration and invasion. The number of migratory cells was 655.00 ± 19.80 *vs*. 214.00 ± 14.35 (*p* < 0.001) and 406.00 ± 13.93 *vs*. 179.00 ± 12.33 (*p* < 0.001) in AGS and N87 cells, before and after re-expression of Lnc21q22.11, respectively (Figure 3(b)). The migratory cells were 207.00 ± 8.83 *vs*. 533.00 ± 14.76 (*p* < 0.001) before and after knockdown of Lnc21q22.11 in BGC-823 cells (Figure 3(b)). The reduced cell numbers by Lnc21q22.11 imply its inhibitory role in cell migration.

The invasive cells were 474.00 ± 20.05 *vs*. 211.00 ± 19.03 (*p* < 0.001) and 355.00 ± 18.71 *vs*. 193.00 ± 11.05 (*p* < 0.001), in Lnc21q22.11 unexpressed and re-expressed AGS and N87 cells, respectively (Figure 3(c)). The invasive cells were 210.00 ± 8.83 *vs*. 384.00 ± 16.06 (*p* < 0.001) in BGC-823 cells expression and knockdown of Lnc21q22.11 (Figure 3(c)). The inhibitory roles of Lnc21q22.11 in cell migration and invasion were validated by examining related proteins. As shown in Figure 3(d), the levels of MMP2, MMP7, and MMP9 were reduced by Lnc21q22.11 in GC cells.

### Lnc21q22.11 restrains GG cell xenograft growth in mice

To explore the role of Lnc21q22.11 *in vivo*, GC cell xenograft model was employed. The tumour volumes were 533.35 ± 85.28 mm^3^ *vs*. 46.94 ± 12.92 mm^3^ in Lnc21q22.11 unexpressed and re-expressed N87 cell xenografts (*p* < 0. 001, Figure 3(e,f)). The tumour weights were 0.302 ± 0.057 g *vs*. 0.013 ± 0.003 g in the Lnc21q22.11 unexpressed and re-expressed N87 cell xenografts, respectively (*p* < 0.001, Figure 3(f)). Tumour volume and weight were significantly reduced by re-expression of Lnc21q22.11.

### Lnc21q22.11 inhibits MEK/ERK signaling pathway via interacting with MYH9 in GC cells

**Figure 4.**
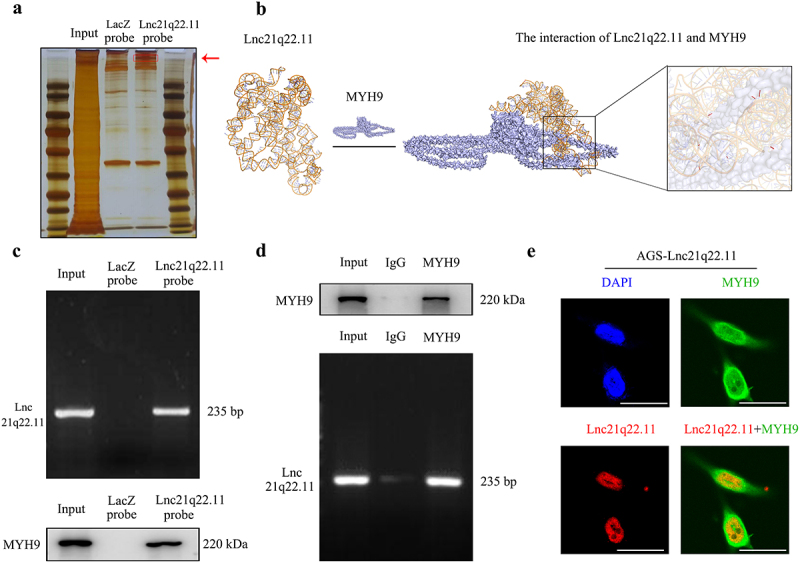
Interaction of Lnc21q22.11 and MYH9, and their co-localization in the nucleus. (a) RNA pull-down assay showing Lnc21q22.11 and protein complexes. The red arrow indicates the differential band. LacZ probes were used for the control group. (b) Predicted interaction between Lnc21q22.11 and MYH9 using Alphafold3. Lnc21q22.11 and MYH9 are represented by ribbon and surface, respectively. Short red bands indicate hydrogen bonds between Lnc21q22.11 and MYH9. (c) Interaction between Lnc21q22.11 and MYH9 verified by RNA pull-down and western blotting using labeled Lnc21q22.11 probes and MYH9 antibodies. (d) Interaction between Lnc21q22.11 and MYH9 validated by RNA immunoprecipitation assay. IgG antibody was used for the control group. (e) Confocal laser microscopic images showing the localization of Lnc21q22.11 (red) and MYH9 (green) in cells, and their co-localization in the nucleus. The scale bar represents 25 μm.

The mechanism was explored to find the connection of Lnc21q22.11 to cancer-related signaling pathways for developing novel therapeutic strategies. Lnc21q22.11 probes were labeled with biotin, and RNA pull-down assay was performed using cell lysates from Lnc21q22.11 highly expressed BGC-823, and re-expressed AGS and N87 cells. By analysing the Lnc21q22.11 binding protein complexes, MYH9 was found to have the highest score and was present in all three cell lines (Figure 4(a), Supplementary figure S4). The interaction between Lnc21q22.11 and MYH9 was also predicted by Alphafold3 (Figure 4(b), https://alphafoldserver.com/). Then the interaction between Lnc21q22.11 and MYH9 was verified by RNA pull-down assay and western blotting, as well as native RIP assay (Figure 4(c,d)). Thereafter, the combination of Lnc21q22.11 and MYH9 was revealed by observing their co-localization in the nucleus in Lnc21q22.11 over-expressed AGS cells (Figure 4(e)).

**Figure 5.**
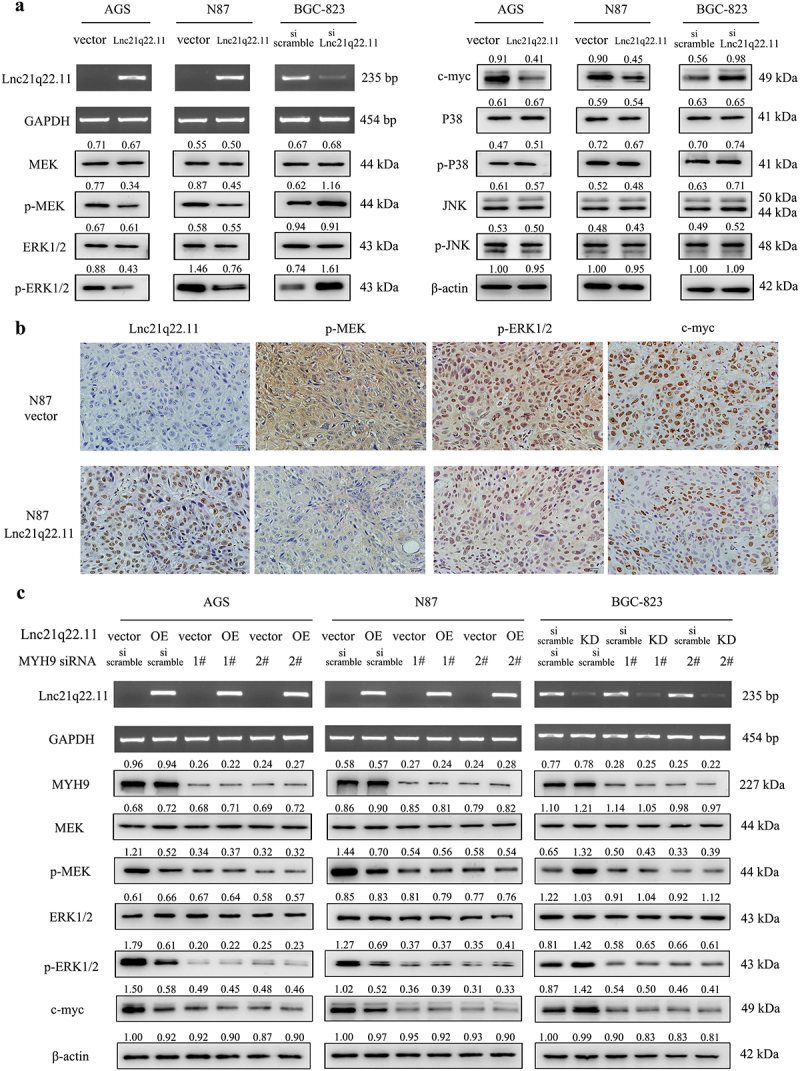
Lnc21q22.11 inhibits the MEK/ERK signaling pathway through interacting with MYH9. (a) Levels of Lnc21q22.11 and key components of the MAPK signaling pathway (MEK, p-MEK, ERK1/2, p-ERK1/2, c-myc, P38, p-P38, JNK and p-JNK) in AGS, N87, and BGC-823 cells. GAPDH: internal control for RT-PCR; β-acin: internal control for western blotting. (b) Levels of Lnc21q22.11 and staining density of p-MEK, p-ERK1/2, c-myc in N87 cell xenografts. (c) Influence of Lnc21q22.11 on the MAPK signaling pathway before and after MYH9 knockdown. OE: overexpression; KD: knockdown.

The biological functions of MYH9 are complex. It plays a crucial role in cancer development and progression [18]. In the interacting complex of Lnc21q22.11 in GC cells, the higher scoring proteins were mainly involved in the mitogen-activated protein kinase (MAPK) signaling pathway (Supplementary table S2). MYH9 has been reported to play key roles in cancer through MAPK signaling pathway [19,20]. Therefore, the role of Lnc21q22.11 in MAPK signaling pathway was explored in GC cells. As shown in Figure 5(a), the levels of p-MEK, p-ERK1/2, and c-myc were reduced by re-expressing Lnc21q22.11 in GC cells, while no obvious difference was observed in p-P38 or p-JNK, indicating that Lnc21q22.11 suppressed MEK/ERK signaling pathway. The effect of Lnc21q22.11 on the MEK/ERK signaling pathway was verified *in vivo* by immunohistochemical staining (Figure 5(b)). Thereafter, siRNA technique was used. After testing efficiency, siMYH9–1# and siMYH9–2# were selected for further experiments (Supplementary figure S5). As shown in Figure 5(c), MYH9 protein levels were unchanged in GC cells after the re-expression or knockdown of Lnc21q22.11. Furthermore, knocking down MYH9 eliminated the difference in MEK/ERK signaling pathway induced by Lnc21q22.11, as evidenced by the reduction of p-MEK, p-ERK1/2, and c-myc to comparable levels after knocking down MYH9 in GC cells (Figure 5(c)). These results demonstrate that although Lnc21q22.11 does not modulate MYH9 expression, it suppresses MEK/ERK signaling pathway by the interaction with MYH9.

### Loss of/reduced Lnc21q22.11 expression sensitizes GC cells to MEK inhibitor

**Figure 6.**
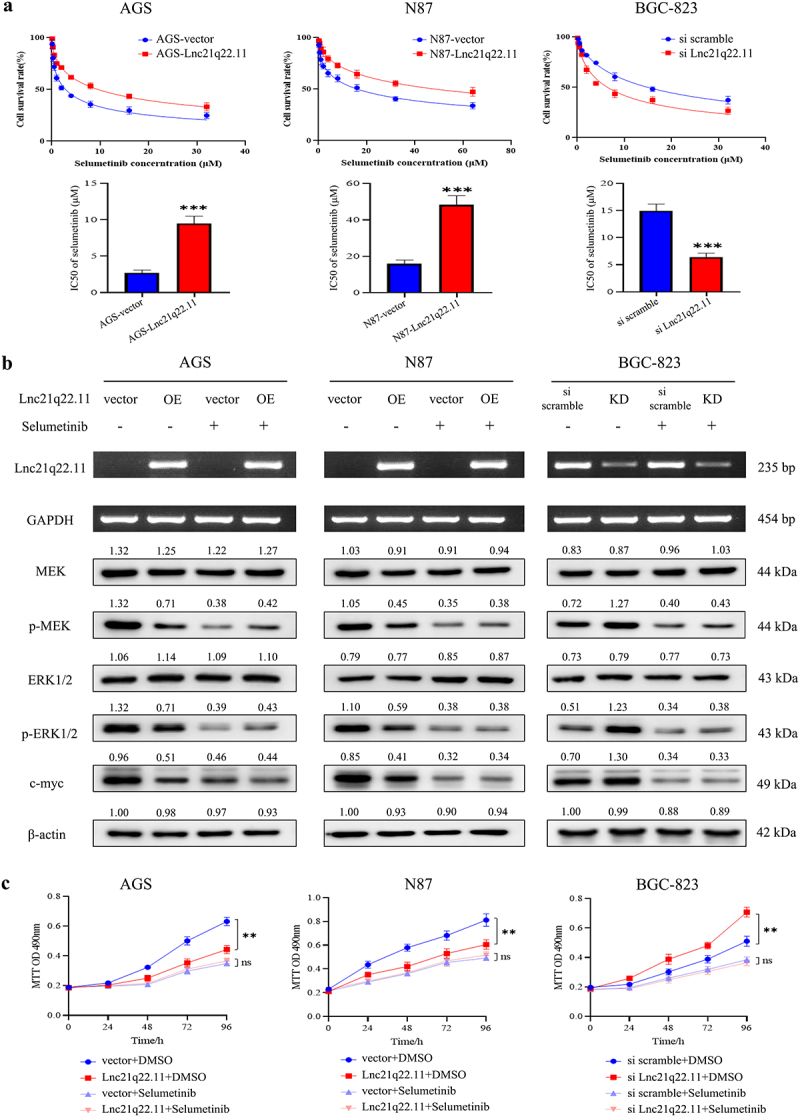
Sensitivity of GC cells to MEK inhibitor with or without Lnc21q22.11 expression. (a) MTT assay showing the half maximal inhibitory concentration of selumetinib in GC cells. (b) Levels of key components in the MEK/ERK signaling pathway with or without Lnc21q22.11 expression in selumetinib treated and untreated GC cells for 48 h (1.5 μM in AGS, 8 μM in N87 and 3 μM in BGC-823 cells). OE: overexpression; KD: knockdown. (c) Sensitivity of GC cells to selumetinib after treatment for 24, 48, 72, and 96 h.

As loss or reduction of Lnc21q22.11 expression activated MEK/ERK signaling pathway, the sensitivity of GC cells with or without Lnc21q22.11 expression to selumetinib, a MEK inhibitor, was assessed by MTT assay. Before and after re-expression of Lnc21q22.11, the half maximal inhibitory concentration (IC_50_) values were 2.71 ± 0.31 μM *vs*. 9.51 ± 0.80 μM (*p* < 0.001) and 15.90 ± 1.65 μM *vs*. 48.35 ± 4.06 μM (*p* < 0.001) in AGS and N87 cells, respectively. In Lnc21q22.11 highly expressed BGC-823 cells, the IC_50_ values were 14.92 ± 1.04 μM *vs*. 6.43 ± 0.57 μM (*p* < 0.001) before and after knockdown of Lnc21q22.11 (Figure 6(a)), demonstrating that loss of/reduced Lnc21q22.11 expression sensitized GC cells to selumetinib. Then, approximately 1/2 IC_50_ concentration of selumetinib was selected for further experiments. Under selumetinib treatment, reduced levels of p-MEK, p-ERK1/2, and c-myc were observed in Lnc21q22.11 unexpressed/reduced GC cells (Figure 6(b)). After treatment for 24, 48, 72, and 96 h, the increase in sensitivity to MEK inhibitor in Lnc21q22.11 unexpressed/reduced GC cells was greater than the increase in Lnc21q22.11 re-expressed/highly expressed GC cells (Figure 6(c)).

## Discussion

MicroRNAs (miRNAs) and lncRNAs are two major types of non-coding RNAs transcribed from a large part of the genome and play important roles in gene expression and protein function. Dysregulation of these transcripts has been linked to various diseases including cancer [21,22]. However, the application of RNA-based therapeutics has been hampered owing to their non-specificity and side effects [15,23]. For example, the phase 1 clinical study of MRX34 (a miR-34 mimic) was terminated due to severe immune-related side effects that led to the deaths of four patients [10,24]. Some clinical trials have been terminated for a lack of efficacy, due to the sequence similarities to endogenous RNAs, instability of ‘naked’ RNA, and the absence of delivery vehicles for targeting specific organs or cell types [15,25,26]. A typical example is that targeting BCL2 mRNA showed limited therapeutic effects, whereas venetoclax, a small molecular inhibitor of the BCL2 protein, has achieved clinical success [15,27,28]. Therefore, the major obstacle of RNA-based cancer therapy is the incomplete understanding of lncRNA molecular biology and the downstream gene regulatory networks for the development of precision therapy [15,23].

Non-coding RNAs may play a dual role in cancer as either oncogenes or tumour suppressors in different cell types. In this study, we identified a novel lncRNA and designated it as Lnc21q22.11 according to the encoding region in the human genome. The full-length sequence was obtained, and its expression was reduced in human gastric cancer. It was mainly localized in the nucleus. The non-protein coding nature of Lnc21q22.11 was verified by the experiments of constructing full-length or predicted ORF fusion expression vectors. The expression of Lnc21q22.11 is modulated by histone modification. Functional studies suggest that Lnc21q22.11 inhibits GC cell proliferation, colony formation, migration, invasion, induces G1/S arrest and cell apoptosis. Lnc21q22.11 suppresses the growth of N87 cell xenografts in mice. The interaction between Lnc21q22.11 and MYH9 was initially identified by RNA pulldown assay and mass spectrometry analysis, and subsequently confirmed by western blot. Further validation by RNA immunoprecipitation revealed the interaction between Lnc21q22.11 and MYH9. MYH9 is a multifunctional protein involved in cancer cell proliferation and metastasis, acting as either an oncogene or tumour suppressor in a context-dependent manner [19,29]. The regulation network of MYH9 is complex, including Wnt/β-catenin, PI3K/AKT, ERK, DNA damage repair, and other signaling pathways [17,30–32]. MYH9 exhibits diverse subcellular localizations, with distinct functions associated with its presence at the cell membrane, cytoplasm, or nucleus [33–35]. Consistent with prior reports about MYH9 in GC, we observed predominant nuclear as well as cytoplasmic localization of MYH9, and the co-localization of Lnc21q22.11 and MYH9 in the nucleus, indicating the interaction between Lnc21q22.11 and nuclear MYH9 [34,36]. Moreover, dynamic cellular localizations of MEK and ERK were reported to critically determine the specificity of signaling regulation [37]. While predominantly cytoplasmic in resting cells, both MEK and ERK undergo a shuttle in and out of the nucleus after ligand stimulation [38]. It was recognized that cell proliferation is mediated mainly by nuclear ERK activity, whereas other cellular processes are mediated by cytoplasmic ERK activity [39,40]. Our study demonstrates that Lnc21q22.11 suppresses gastric cancer cell growth by inhibiting MEK/ERK signaling pathway via the interaction with MYH9 in the nucleus.

RNA-based cancer therapy has experienced a critical challenge in clinical translation. Targeting downstream signaling pathways is a more viable approach to avoid immune-mediated toxicity and off-target effects. Therefore, there is an urgent need to completely understand the biological functions and gene regulatory networks of these lncRNAs. The application of the synthetic lethality principle in cancer therapy provides a model for developing therapeutic strategies based on RNA abnormalities. In addition to targeting downstream pathways, the partner pathways of synthetic lethality will be uncovered by a comprehensive understanding of the gene regulatory networks. Epigenetic-based ‘synthetic lethality’ has been a successful therapeutic strategy for cancers with the silencing of untargetable lncRNAs [41,42].

In conclusion, Lnc21q22.11 is a novel long non-coding RNA, and its expression is reduced in gastric cancer. The expression of Lnc21q22.11 is regulated by histone modification. Lnc21q22.11 suppresses GC growth by inhibiting the MEK/ERK signaling pathway both *in vitro* and *in vivo* (GraphicalAbstract). Loss of/reduced Lnc21q22.11 expression sensitizes GC cells to MEK inhibitor.

## Supplementary Material

Supplemental Material

## Data Availability

The raw RNA sequencing data have been deposited in the Genome Sequence Archive for Human (GSA for Human, https://ngdc.cncb.ac.cn/gsa-human/), the accession number is HRA011214. Other raw data for the study are available in Supplementary materials.

## References

[1] Yeoh K, Tan P. G and P tan mapping the genomic diaspora of gastric cancer. Nat Rev Cancer. 2022;22(2):71–16. doi: 10.1038/s41568-021-00412-7 34702982

[2] Smyth EC, Nilsson M, Grabsch HI, et al. Gastric cancer. Lancet. 2020;396(10251):635–648. doi: 10.1016/s0140-6736(20)31288-5 32861308

[3] Oliveira C, Pinheiro H, Figueiredo J, et al. Familial gastric cancer: genetic susceptibility, pathology, and implications for management. Lancet Oncol. 2015;16(2):e60–70. doi: 10.1016/s1470-2045(14)71016-2 25638682

[4] Usui G, Matsusaka K, Huang KK, et al. Integrated environmental, lifestyle, and epigenetic risk prediction of primary gastric neoplasia using the longitudinally monitored cohorts. EBioMedicine. 2023;98:104844. doi: 10.1016/j.ebiom.2023.104844 38251469 PMC10755115

[5] Joshi SS, D B. Badgwell current treatment and recent progress in gastric cancer. CA Cancer J Clin. 2021;71(3):264–279. doi: 10.3322/caac.21657 33592120 PMC9927927

[6] Nakamura Y, Kawazoe A, Lordick F, et al. Biomarker-targeted therapies for advanced-stage gastric and gastro-oesophageal junction cancers: an emerging paradigm. Nat Rev Clin Oncol. 2021;18(8):473–487. doi: 10.1038/s41571-021-00492-2 33790428

[7] Dlamini Z, Ladomery MR, Kahraman Editorial A. Editorial: the RNA revolution and cancer. Front Endocrinol (Lausanne). 2024;15:1422599. doi: 10.3389/fendo.2024.1422599 38832352 PMC11144892

[8] Brannan CI, Dees EC, Ingram RS, et al. The product of the H19 gene may function as an RNA. Mol Cell Biol. 1990;10(1):28–36. doi: 10.1128/MCB.10.1.28 1688465 PMC360709

[9] Brown CJ, Ballabio A, Rupert JL, et al. A gene from the region of the human X inactivation centre is expressed exclusively from the inactive X chromosome. Nature. 1991;349(6304):38–44. doi: 10.1038/349038a0 1985261

[10] Nemeth K, Bayraktar R, Ferracin M, et al. Non-coding RNAs in disease: from mechanisms to therapeutics. Nat Rev Genet. 2024;25(3):211–232. doi: 10.1038/s41576-023-00662-1 37968332

[11] Frankish A, Diekhans M, Ferreira AM, et al. GENCODE reference annotation for the human and mouse genomes. Nucleic Acids Res. 2019;47(D1):D766–d773. doi: 10.1093/nar/gky955 30357393 PMC6323946

[12] Zhao L, Wang J, Li Y, et al. NONCODEV6: an updated database dedicated to long non-coding RNA annotation in both animals and plants. Nucleic Acids Res. 2021;49(D1):D165–d171. doi: 10.1093/nar/gkaa1046 33196801 PMC7779048

[13] Pennisi E. Surprise RNA paints colorful patterns on butterfly wings. Science. 2024;383(6687):1039–1040. doi: 10.1126/science.adp0471 38452086

[14] Kaur R, McGarry A, Shropshire JD, et al. Prophage proteins alter long noncoding RNA and DNA of developing sperm to induce a paternal-effect lethality. Science. 2024;383(6687):1111–1117. doi: 10.1126/science.adk9469 38452081 PMC11187695

[15] Winkle M, El-Daly SM, Fabbri M, et al. Noncoding RNA therapeutics - challenges and potential solutions. Nat Rev Drug Discov. 2021;20(8):629–651. doi: 10.1038/s41573-021-00219-z 34145432 PMC8212082

[16] Herman JG, Graff JR, Myöhänen S, et al. Methylation-specific PCR: a novel PCR assay for methylation status of CpG islands. Proc Natl Acad Sci USA. 1996;93(18):9821–9826. doi: 10.1073/pnas.93.18.9821 8790415 PMC38513

[17] Du Q, Zhang M, Gao A, et al. Epigenetic silencing ZSCAN23 promotes pancreatic cancer growth by activating wnt signaling. Cancer Biol Ther. 2024;25(1):2302924. doi: 10.1080/15384047.2024.2302924 38226836 PMC10793710

[18] Li Y, Pan Y, Yang X, et al. Unveiling the enigmatic role of MYH9 in tumor biology: a comprehensive review. Cell Commun Signal. 2024;22(1):417. doi: 10.1186/s12964-024-01781-w 39192336 PMC11351104

[19] Wang B, Qi X, Liu J, et al. MYH9 promotes growth and metastasis via activation of MAPK/AKT signaling in colorectal cancer. J Cancer. 2019;10(4):874–884. doi: 10.7150/jca.27635 30854093 PMC6400792

[20] You GR, Chang JT, Li YL, et al. MYH9 facilitates cell invasion and radioresistance in head and neck cancer via modulation of cellular ROS levels by activating the MAPK-Nrf2-GCLC pathway. Cells. 2022;11(18):2855. doi: 10.3390/cells11182855 36139430 PMC9497050

[21] Srinivasan S, Yeri A, Cheah PS, et al. Small RNA sequencing across diverse biofluids identifies optimal methods for exRNA isolation. Cell. 2019;177(2):446–462.e16. doi: 10.1016/j.cell.2019.03.024 30951671 PMC6557167

[22] Vancura A, Gutierrez AH, Hennig T, et al. Is evolutionary conservation a useful predictor for cancer long noncoding RNAs? Insights from the cancer LncRNA census 3. Noncoding RNA. 2022;8(6). doi: 10.3390/ncrna8060082 PMC9785742 36548181

[23] Coan M, Haefliger S, Ounzain S, et al. Targeting and engineering long non-coding RNAs for cancer therapy. Nat Rev Genet. 2024;25(8):578–595. doi: 10.1038/s41576-024-00693-2 38424237

[24] Hong DS, Kang YK, Borad M, et al. Phase 1 study of MRX34, a liposomal miR-34a mimic, in patients with advanced solid tumours. Br J Cancer. 2020;122(11):1630–1637. doi: 10.1038/s41416-020-0802-1 32238921 PMC7251107

[25] Warren MS, Hughes SG, Singleton W, et al. Results of a proof of concept, double-blind, randomized trial of a second generation antisense oligonucleotide targeting high-sensitivity C-reactive protein (hs-CRP) in rheumatoid arthritis. Arthritis Res Ther. 2015;17(1):80. doi: 10.1186/s13075-015-0578-5 25885521 PMC4415222

[26] Nguyen QD, Schachar RA, Nduaka CI, et al. Dose-ranging evaluation of intravitreal siRNA PF-04523655 for diabetic macular edema (the DEGAS study). Invest Ophthalmol Vis Sci. 2012;53(12):7666–7674. doi: 10.1167/iovs.12-9961 23074206

[27] Moulder SL, Symmans WF, Booser DJ, et al. Phase I/II study of G3139 (Bcl-2 antisense oligonucleotide) in combination with doxorubicin and docetaxel in breast cancer. Clinical Cancer Res. 2008;14(23):7909–7916. doi: 10.1158/1078-0432.Ccr-08-1104 19047121 PMC4375958

[28] Croce CM, Reed JC. Finally, an apoptosis-targeting therapeutic for cancer. Cancer Research. 2016;76(20):5914–5920. doi: 10.1158/0008-5472.Can-16-1248 27694602 PMC5117672

[29] Liu Q, Cheng C, Huang J, et al. MYH9: a key protein involved in tumor progression and virus-related diseases. Biomed Pharmacother. 2024;171:116118. doi: 10.1016/j.biopha.2023.116118 38181716

[30] Kai JD, Cheng LH, Li BF, et al. MYH9 is a novel cancer stem cell marker and prognostic indicator in esophageal cancer that promotes oncogenesis through the PI3K/AKT/mTOR axis. Cell Biology International. 2022;46(12):2085–2094. doi: 10.1002/cbin.11894 36030536

[31] Gao S, Wang S, Zhao Z, et al. TUBB4A interacts with MYH9 to protect the nucleus during cell migration and promotes prostate cancer via GSK3β/β-catenin signalling. Nat Commun. 2022;13(1):2792. doi: 10.1038/s41467-022-30409-1 35589707 PMC9120517

[32] Wang G, Huang W, Li W, et al. TFPI-2 suppresses breast cancer cell proliferation and invasion through regulation of ERK signaling and interaction with actinin-4 and myosin-9. Sci Rep. 2018;8(1):14402. doi: 10.1038/s41598-018-32698-3 30258071 PMC6158255

[33] Vicente-Manzanares M, Ma X, Adelstein RS, et al. Non-muscle myosin II takes centre stage in cell adhesion and migration. Nat Rev Mol Cell Biol. 2009;10(11):778–790. doi: 10.1038/nrm2786 19851336 PMC2834236

[34] Ye G, Yang Q, Lei X, et al. Nuclear MYH9-induced CTNNB1 transcription, targeted by staurosporin, promotes gastric cancer cell anoikis resistance and metastasis. Theranostics. 2020;10(17):7545–7560. doi: 10.7150/thno.46001 32685004 PMC7359096

[35] Arii J, Goto H, Suenaga T, et al. Non-muscle myosin IIA is a functional entry receptor for herpes simplex virus-1. Nature. 2010;467(7317):859–862. doi: 10.1038/nature09420 20944748

[36] Ye G, Huang K, Yu J, et al. MicroRNA-647 targets SRF-MYH9 axis to suppress invasion and metastasis of gastric cancer. Theranostics. 2017;7(13):3338–3353. doi: 10.7150/thno.20512 28900514 PMC5595136

[37] Maik-Rachline G, Hacohen-Lev-Ran A, Seger Nuclear R. Nuclear ERK: mechanism of translocation, substrates, and role in cancer. Int J Mol Sci. 2019;20(5):1194. doi: 10.3390/ijms20051194 30857244 PMC6429060

[38] Zehorai E, Yao Z, Plotnikov A, et al. The subcellular localization of MEK and ERK–a novel nuclear translocation signal (NTS) paves a way to the nucleus. Mol Cell Endocrinol. 2010;314(2):213–220. doi: 10.1016/j.mce.2009.04.008 19406201

[39] Michailovici I, Harrington HA, Azogui HH, et al. Nuclear to cytoplasmic shuttling of ERK promotes differentiation of muscle stem/progenitor cells. Development. 2014;141(13):2611–2620. doi: 10.1242/dev.107078 24924195 PMC4067960

[40] Formstecher E, Ramos JW, Fauquet M, et al. PEA-15 mediates cytoplasmic sequestration of ERK MAP kinase. Dev Cell. 2001;1(2):239–250. doi: 10.1016/s1534-5807(01)00035-1 11702783

[41] Gao A, Guo M. Epigenetic based synthetic lethal strategies in human cancers. Biomark Res. 2020;8(1):44. doi: 10.1186/s40364-020-00224-1 32974031 PMC7493427

[42] Liu F, Gao A, Zhang M, et al. Methylation of FAM110C is a synthetic lethal marker for ATR/CHK1 inhibitors in pancreatic cancer. J Transl Int Med. 2024;12(3):274–287. doi: 10.2478/jtim-2023-0128 39081276 PMC11284899

