# Supplementary material for: A novel lncRNA, Lnc21q22.11, suppresses gastric cancer growth by inhibiting MEK/ERK pathway
Source: Epigenetics. 2025 Jun 2;20(1):2512764. doi: 10.1080/15592294.2025.2512764 (PMC12140449; doi:10.1080/15592294.2025.2512764)
Supplement: Supplemental Material [file KEPI_A_2512764_SM2130.zip › Supplementary files/Supplementary legends.docx]

**Supplementary figure 1** The structure of Lnc21q22.11 gene, and expression levels of Lnc21q22.11 and its neighboring genes.

(a) Lnc21q22.11 gene contains two exons and one intron. Exon1 is 556 bp, exon2 is 646 bp, and the intron is 4074 bp in length. Lnc21q22.11 is partially overlapped with the neighboring genes KCNE2, SMIM11, and LOC105372791. TSS: transcriptional start site; Lnc21q22.11-F: forward RT-PCR primer for Lnc21q22.11; Lnc21q22.11-R: reverse RT-PCR primer for Lnc21q22.11. (b) Real-time PCR result showed no significant change in the expression levels of KCNE2, SMIM11, and LOC105372791 by Lnc21q22.11 in AGS, N87, and BGC-823 cells. ****p* < 0.001; ns: no significant difference.

**Supplementary figure 2** The non-coding entity of Lnc21q22.11.

(a) Potential open reading frames (ORFs) of Lnc21q22.11 predicted by ORF Finder. ORF2 and ORF3 showed consistent transcription direction (dark blue background). (b) Fusion protein constructs for ORF2/ORF3 were built with GFP whose ATG at 5’ end was mutated into ATT. Wild type GFP served as a positive control. (c) Successful transfection of ORF2-mutGFP, ORF3-mutGFP, and wild type GFP vectors was validated by RT-PCR in AGS cells. (d) Results of fusion protein expression.

**Supplementary figure 3** Expression of Lnc21q22.11 and methylation status of its promoter region detected by methylation-specific PCR.

U: unmethylation; M: methylation; IVD: in vitro methylated DNA, methylated control; NL: normal peripheral lymphocytes DNA, unmethylated control; H_2_O: negative control.

**Supplementary figure 4** Overlapping proteins obtained from RNA pull-down assay followed by mass spectrum analysis.

**Supplementary figure 5** Efficiency of siRNAs in knocking down MYH9.

**Table legends**

**Table 1** Association between Lnc21q22.11 expression and clinical-pathological characteristics of GC patients.

*P* values were obtained from χ^2^ test, **p* < 0.05.

**Supplementary table 1** The list of probe/primer/siRNA sequences.

**Supplementary table 2** Analysis of results of RNA-pull down and mass spectrometry.

Note: Proteins whose scores were less than 2 were omitted in analysis.
