# Supplementary material for: A novel lncRNA, Lnc21q22.11, suppresses gastric cancer growth by inhibiting MEK/ERK pathway
Source: Epigenetics. 2025 Jun 2;20(1):2512764. doi: 10.1080/15592294.2025.2512764 (PMC12140449; doi:10.1080/15592294.2025.2512764)
Supplement: Supplemental Material [file KEPI_A_2512764_SM2130.zip › Supplementary files/Supplementary_table_1.docx]

Supplementary table 1 The list of probe/primer/siRNA sequences

| **Probes/primers/siRNA sequence** | |
| --- | --- |
| Lnc21q22.11 original sequence | CGGGCAGCCCTGGCCAGCAGCTCTTGTCCCTGTGGCTGGAGGCTGAAGTCCACGTAGGCCCCGGCGGGGAGCCGCTGGGGTGTAGGCCGGGTGCCTTTGTCCAAGCCTGGCGCCACTGCCCAGACACTTTACTTCAAATTTGGCTTTATTTTCATCATTGATATTGATTAAAATGACATTCACTTGCCCGGAAAGTAAAAGAGGTCTTCAGCACAAAATATTGCTCCAACAAGCAAGCATAAATCACTGAGATTGAAATGTCATCTATCTTTATTGGCCAACCACATAATCTCCATGAAAATTCTCAACAGAGAGCAAGGAACTCACTTTCTTCTAAGCAAAGACAATTTGGATTTGCCTCGTGGCACTGGCATCTCTTCATGTCTGGACGTCAGATGTTAGCTTGGTGCCTTTCTCCCTTATCAGGGGGACATTTTGAACCCAGCCGCACCAATGTTCTCATGGATGGTG |
| Lnc21q22.11 northern blot probe | GGTCCCGAAATGCACCGCTACGTGCTCAGGCGCAGTGGGCCGGTGCCGCCGACGAGAGTGCACTACTCCGTGCGCAGGCGCAGTGGGCCCGGGCAGAAGACCT |
| GAPDH northern blot probe | GTTCGTCATGGGTGTGAACCATGAGAAGTATGACAACAGCCTCAAGATCATCAGCAATGCCTCCTGCACCACCAACTGCTTAGCACCCCTGGCCAAGGTCATCCATGACAACTTTGGTATCGTGGAAGGACTCATGACCACAGTCC |
| Lnc21q22.11 full-length sequence | GCTCAACGGGTTGACGGCGCCCGACGGCCAGGTCCAGCAGGGGCGCCCCGCAGGTGGGGCCTGGCGGCCTTCACCTTCCGGATCCCCGACCCGGCGCGACCGGGAGCCGGCAGACTTTTGTCTAGGAGGGAAAACCCGAGCGCGGGGCCGGCCGCGACATCGCAGCATCCCAAAGAGCTTTTGGAATTTGGGTCACTCCCTTCTACCCCGACGTCACACACTTACTGCTCCCGGCAGCGGAGGCTCCAGCGCCTGGCCGCGCACAAACCACGACTTCTACCGTCCTGCCGGGGAAAACTACAGGTCCCGAAATGCACCGCTACGTGCTCAGGCGCAGTGGGCCGGTGCCGCCGACGAGAGTGCACTACTCCGTGCGCAGGCGCAGTGGGCCCGGGCAGAAGACCTGGGGCGCGCTGCTCACTGCGCAGGCGCAGTGAGCCCAGGCGGGCAGCCCTGGCCAGCAGCTCTTGTCCCTGTGGCTGGAGGCTGAAGTCCACGTAGGCCCCGGCGGGGAGCCGCTGGGGTGTAGGCCGGGTGCCTTTGTCCAAGCCTGGCGCCACTGCCCAGACACTTTACTTCAAATTTGGCTTTATTTTCATCATTGATATTGATTAAAATGACATTCACTTGCCCGGAAAGTAAAAGAGGTCTTCAGCACAAAATATTGCTCCAACAAGCAAGCATAAATCACTGAGATTGAAATGTCATCTATCTTTATTGGCCAACCACATAATCTCCATGAAAATTCTCAACAGAGAGCAAGGAACTCACTTTCTTCTAAGCAAAGACAATTTGGATTTGCCTCGTGGCACTGGCATCTCTTCATGTCTGGACGTCAGATGTTAGCTTGGTGCCTTTCTCCCTTATCAGGGGGACATTTTGAACCCAGCCGCACCAATGTTCTCATGGATGGTGGCCTTCGATTCTTCTAGATTCAAGATTTGGCTCTTGTACTTTTCCTGCCAGTCCTCTACAATGTACTGGTGGTAGGGGTCATTGGAGTGTTCCCGTCTCTTGGATTTCACAGTGCTCACCAGGATGGCCACGATGATGAAAGAGAACATTCCAATCATCACCATGAGGTACAGGATGACATAGTAGAAGTTCTCAGCATCAACTTTGGCTTGGAGGGCCTCTTGCTCAGCTGTTGTGTTCTGGCGCCAATTGTCCATATAAGTAATAAAAATCCTTCGGAAGACGTC |
| Lnc21q22.11 RNA pull down and FISH probes |  |
| probe1 | AAGTGAGTTCCTTGCTCTCTGTTGAG |
| probe2 | CGGGGCCTACGTGGACTTCAGCCTCCAGCC |
| probe3 | GTACCTCATGGTGATGATTGGAATGTTCTC |
| probe4 | GCCGGGAGCAGTAAGTGTGTGACGTC |
| probe5 | CAGGACGGTAGAAGTCGTGGT |
| probe6 | ACTGCGCCTGCGCACGGAGTAGTGCAC |
| LacZ probe1 | GATCTTCCAGATAACTGCCG |
| LacZ probe2 | TTAACGCCTCGAATCAGCAA |
| 5'RACE gene specific primer | GGAGCAGTAAGTGTGTGACGTCGG |
| 3'RACE gene specific primer | AGATTCAAGATTTGGCTCTT |
| GFP PCR primer-Forward | ATGGAGAGCGACGAGAGC |
| GFP PCR primer-Reverse | TCTTTCTTCACCGGCATCTGC |
| ORF2-GFP PCR primer-Forward | ATGGACAATTGGCGCCAGAAC |
| ORF2-GFP PCR primer-Reverse | TCTTTCTTCACCGGCATCTGC |
| ORF3-GFP PCR primer-Forward | ATGCTGCGATGTCGCGG |
| ORF3-GFP PCR primer-Reverse | TCTTTCTTCACCGGCATCTGC |
| Lnc21q22.11 PCR primer-Forward | ACCACGACTTCTACCGTCCT |
| Lnc21q22.11 PCR primer-Reverse | CTACGTGGACTTCAGCCTCC |
| KCNE2 PCR primer-Forward | CTGTGGGGATAGAAAGGAGAGA |
| KCNE2 PCR primer-Reverse | TGCTCCTATGTCCGTCTGGT |
| SMIM11 PCR primer-Forward | ACTTGTCTGTTGGGGGCTTG |
| SMIM11 PCR primer-Reverse | TGTTGTTTTGCCTCCAACCT |
| LOC105372791 primer-Forward | CCAGCGTCTGTGTGAAATTGG |
| LOC105372791 primer-Reverse | GCCAAATCCAGAAAAGATCCGT |
| Lnc21q22.11 MSP unmethylation primer-Forward | TTTTGTTTGGGTTATGTGTTAGTTG |
| Lnc21q22.11 MSP unmethylation primer-Reverse | AACAAAAATCTACCAACTCCCAATCA |
| Lnc21q22.11 MSP methylation primer-Forward | TCGTTCGGGTTACGTGTTAGTCG |
| Lnc21q22.11 MSP methylation primer-Reverse | CAAAAATCTACCGACTCCCGATCG |
| CHIP PCR primer-Forward | CTCAGCTCCTCCTGGGTAA |
| CHIP PCR primer-Reverse | GAGTCTAGGGAGACAAGCAATC |
| GAPDH PCR primer-Forward | GACCACAGTCCATGCCATCAC |
| GAPDH PCR primer-Reverse | GTCCACCACCCTGTTGCTGTA |
| U6 PCR primer-Forward | CTCGCTTCGGCAGCACA |
| U6 PCR primer-Reverse | AACGCTTCACGAATTTGCGT |
| GFP PCR primer-Forward | ATGGAGAGCGACGAGAGCGGC |
| GFP PCR primer-Reverse | TCTTTCTTCACCGGCATCTGCATCC |
| ORF-GFP primer-Forward | GCTCAACGGGTTGACGGC |
| ORF-GFP primer-Reverse | TCTTTCTTCACCGGCATCTGC |
| Lnc21q22.11 siRNA |  |
| Sense | CCCAGACACUUUACUUCAATT |
| Antisense | UUGAAGUAAAGUGUCUGGGTT |
| MYH9 siRNA 01 |  |
| Sense | CCAGAAGGCGCAGACUAAATT |
| Antisense | UUUAGUCUGCGCCUUCUGGAC |
| MYH9 siRNA 02 |  |
| Sense | CCAGUCCUCUGACAAGUUUTT |
| Antisense | AAACUUGUCAGAGGACUGGTG |
| MYH9 siRNA 03 |  |
| Sense | GCAAGCUGCCGAUAAGUAUTT |
| Antisense | AUACUUAUCGGCAGCUUGCTG |
| siRNA-scramble sense | UUCUCCGAACGUGUCACGUTT |
| siRNA-scramble antisense | ACGUGACACGUUCGGAGAATT |
