# Supplementary material for: A novel lncRNA, Lnc21q22.11, suppresses gastric cancer growth by inhibiting MEK/ERK pathway
Source: Epigenetics. 2025 Jun 2;20(1):2512764. doi: 10.1080/15592294.2025.2512764 (PMC12140449; doi:10.1080/15592294.2025.2512764)
Supplement: Supplemental Material [file KEPI_A_2512764_SM2130.zip › Supplementary files/Supplementary_table_2.docx]

Supplementary table 2 Analysis of results of RNA-pull down and mass spectrometry

| **Protein** | **Score** | **Related signal pathways** |
| --- | --- | --- |
| MYH9 | 249.73 | **MAPK**-Nrf2-GCLC[1], **MAPK**/AKT[2],GSK3β/β-catenin/c-Jun signaling[3-6], ALK5/Smad2/3[7], NOTCH[8], PI3K/AKT/Mtor[9-12], GRK3-YAP[13], AKT/c-Myc[14] |
| MYH10 | 145.56 | **MAPK**[15, 16],EGFR pathway[17], Wnt/β-catenin[18] |
| TMOD3 | 47.24 | **MAPK**/ERK[19], EGFR-PI3K-AKT[20], SND1/RhoA signaling[21] |
| YBOX1 | 39.06 | **MAPK**[22-25], **ERK** [26], Wnt/β-catenin[27], EGFR[28, 29], EGFR/AKT[30], PI3K/AKT/mTOR[31], NF-κB[32-34], AKT/YB1/HIF1α[35], YBX1/PD-L1[36], AKT/GSK3β[37], STAT3[38], YAP1/Hippo[39] |
| DESP | 21.57 | **MAPK**[40, 41], **ERK1/2** [42], **P38** [43],TGFβ[44] [45], Wnt/β-catenin [46] [47] |
| MYO1E | 18.41 | **ERK** [48],Phosphatidylinositol signaling[49] |
| TIA1 | 18.36 | **MAPK/P38** [50], mTORC1 [51], HIF-1α[52] |
| LAP2A | 18.32 | NF-κB [53], Retinoblastoma-E2F pathway [54], DNA damage repair [55] |
| EF1A1 | 12.90 | **MAPK** **P38/JNK/ERK** [56], AKT/mTOR[57], FAK[58], STAT1-cyclin D1[59], AKT and ERK[60], p53-family signaling[61] |
| HNRNPM | 12.19 | **MAPK/AKT**[62], WNT/β-catenin[63], circPTPN12/hnRNPM/IL-6/STAT3[64], IFN[65], PI3K/AKT/mTOR[66] |
| PABPC1 | 9.41 | NOTCH1[67], PABPC1/PAK1[68], PI3K/AKT[69], PTEN[70] |
| PC | 7.47 | Wnt[71], HIF1α and HIF2α[72], p53[73], c-Myc[74] |
| YBOX3 | 5.54 | YBX3/PCNA/p21[75], PI3K/AKT[76] |
| ANXA2 | 2.30 | **ERK3**[77],YAP[78, 79],Wnt/β-catenin[80],NF-κB[81] |

Note: Proteins whose scores were less than 2 were omitted in analysis

Reference

1. You G R, J T Chang, Y L Li, et al. MYH9 Facilitates Cell Invasion and Radioresistance in Head and Neck Cancer via Modulation of Cellular ROS Levels by Activating the MAPK-Nrf2-GCLC Pathway. Cells. 2022; 11(18). doi: 10.3390/cells11182855.

2. Wang B, X Qi, J Liu, et al. MYH9 Promotes Growth and Metastasis via Activation of MAPK/AKT Signaling in Colorectal Cancer. J Cancer. 2019; 10(4): 874-884. doi: 10.7150/jca.27635.

3. Hou R, Y Li, X Luo, et al. ENKUR expression induced by chemically synthesized cinobufotalin suppresses malignant activities of hepatocellular carcinoma by modulating β-catenin/c-Jun/MYH9/USP7/c-Myc axis. Int J Biol Sci. 2022; 18(6): 2553-2567. doi: 10.7150/ijbs.67476.

4. Li Q, H Luo, F Q Dai, et al. SAMD9 Promotes Postoperative Recurrence of Esophageal Squamous Cell Carcinoma by Stimulating MYH9-Mediated GSK3β/β-Catenin Signaling. Adv Sci (Weinh). 2023; 10(11): e2203573. doi: 10.1002/advs.202203573.

5. Lin X, A M Li, Y H Li, et al. Silencing MYH9 blocks HBx-induced GSK3β ubiquitination and degradation to inhibit tumor stemness in hepatocellular carcinoma. Signal Transduct Target Ther. 2020; 5(1): 13. doi: 10.1038/s41392-020-0111-4.

6. Liu L, Y Ning, J Yi, et al. miR-6089/MYH9/β-catenin/c-Jun negative feedback loop inhibits ovarian cancer carcinogenesis and progression. Biomed Pharmacother. 2020; 125: 109865. doi: 10.1016/j.biopha.2020.109865.

7. Sun X, M Zhu, X Chen, et al. MYH9 Inhibition Suppresses TGF-β1-Stimulated Lung Fibroblast-to-Myofibroblast Differentiation. Front Pharmacol. 2020; 11: 573524. doi: 10.3389/fphar.2020.573524.

8. Yang S B, Z H Zhou, J Lei, et al. TM4SF1 upregulates MYH9 to activate the NOTCH pathway to promote cancer stemness and lenvatinib resistance in HCC. Biol Direct. 2023; 18(1): 18. doi: 10.1186/s13062-023-00376-8.

9. Kai J D, L H Cheng, B F Li, et al. MYH9 is a novel cancer stem cell marker and prognostic indicator in esophageal cancer that promotes oncogenesis through the PI3K/AKT/mTOR axis. Cell Biol Int. 2022; 46(12): 2085-2094. doi: 10.1002/cbin.11894.

10. Chen M, L X Sun, L Yu, et al. MYH9 is crucial for stem cell-like properties in non-small cell lung cancer by activating mTOR signaling. Cell Death Discov. 2021; 7(1): 282. doi: 10.1038/s41420-021-00681-z.

11. Zhao R, Y Ge, Y Gong, et al. NAP1L5 targeting combined with MYH9 Inhibit HCC progression through PI3K/AKT/mTOR signaling pathway. Aging (Albany NY). 2022; 14(22): 9000-9019. doi: 10.18632/aging.204377.

12. Xu Z, M Liu, J Wang, et al. Single-cell RNA-sequencing analysis reveals MYH9 promotes renal cell carcinoma development and sunitinib resistance via AKT signaling pathway. Cell Death Discov. 2022; 8(1): 125. doi: 10.1038/s41420-022-00933-6.

13. Li X, J Jiang, Q Wu, et al. TRIM58 downregulation maintains stemness via MYH9-GRK3-YAP axis activation in triple-negative breast cancer stem cells. Cancer Gene Ther. 2024; 31(8): 1186-1200. doi: 10.1038/s41417-024-00780-w.

14. Liu F, L Peng, and J Xi [High expression of MYH9 inhibits apoptosis of non-small cell lung cancer cells through activating the AKT/c-Myc pathway]. Nan Fang Yi Ke Da Xue Xue Bao. 2023; 43(4): 527-536. doi: 10.12122/j.issn.1673-4254.2023.04.04.

15. Cai Z, S Zhang, P Wu, et al. A novel potential target of IL-35-regulated JAK/STAT signaling pathway in lupus nephritis. Clin Transl Med. 2021; 11(2): e309. doi: 10.1002/ctm2.309.

16. Jiang X, Y Lu, S Xie, et al. miR-624 accelerates the growth of liver cancer cells by inhibiting EMC3. Noncoding RNA Res. 2023; 8(4): 641-644. doi: 10.1016/j.ncrna.2023.09.005.

17. Jin Q, M Cheng, X Xia, et al. Down-regulation of MYH10 driven by chromosome 17p13.1 deletion promotes hepatocellular carcinoma metastasis through activation of the EGFR pathway. J Cell Mol Med. 2021; 25(24): 11142-11156. doi: 10.1111/jcmm.17036.

18. Wang Y, Q Yang, Y Cheng, et al. Myosin Heavy Chain 10 (MYH10) Gene Silencing Reduces Cell Migration and Invasion in the Glioma Cell Lines U251, T98G, and SHG44 by Inhibiting the Wnt/β-Catenin Pathway. Med Sci Monit. 2018; 24: 9110-9119. doi: 10.12659/msm.911523.

19. Jin C, Z Chen, W Shi, et al. Tropomodulin 3 promotes liver cancer progression by activating the MAPK/ERK signaling pathway. Oncol Rep. 2019; 41(5): 3060-3068. doi: 10.3892/or.2019.7052.

20. Zheng H, Y Yang, Y G Hong, et al. Tropomodulin 3 modulates EGFR-PI3K-AKT signaling to drive hepatocellular carcinoma metastasis. Mol Carcinog. 2019; 58(10): 1897-1907. doi: 10.1002/mc.23083.

21. Chen B, M Wang, J Qiu, et al. Cleavage of tropomodulin-3 by asparagine endopeptidase promotes cancer malignancy by actin remodeling and SND1/RhoA signaling. J Exp Clin Cancer Res. 2022; 41(1): 209. doi: 10.1186/s13046-022-02411-4.

22. Zeng W, Y Pan, H Chen, et al. YBX1, Targeted By Microrna-382-5p, Promotes Laryngeal Squamous Cell Carcinoma Progression via Modulating RAS/MAPK Signaling. Recent Pat Anticancer Drug Discov. 2024; 19(2): 176-187. doi: 10.2174/1574892818666230207091720.

23. Wu L, S Huang, W Tian, et al. PIWI-interacting RNA-YBX1 inhibits proliferation and metastasis by the MAPK signaling pathway via YBX1 in triple-negative breast cancer. Cell Death Discov. 2024; 10(1): 7. doi: 10.1038/s41420-023-01771-w.

24. Ali M M, V S Akhade, S T Kosalai, et al. PAN-cancer analysis of S-phase enriched lncRNAs identifies oncogenic drivers and biomarkers. Nat Commun. 2018; 9(1): 883. doi: 10.1038/s41467-018-03265-1.

25. Roßner F, C Gieseler, M Morkel, et al. Uncoupling of EGFR-RAS signaling and nuclear localization of YBX1 in colorectal cancer. Oncogenesis. 2016; 5(1): e187. doi: 10.1038/oncsis.2015.51.

26. Jayavelu A K, T M Schnöder, F Perner, et al. Splicing factor YBX1 mediates persistence of JAK2-mutated neoplasms. Nature. 2020; 588(7836): 157-163. doi: 10.1038/s41586-020-2968-3.

27. Wang J, D Shen, S Li, et al. LINC00665 activating Wnt3a/β-catenin signaling by bond with YBX1 promotes gastric cancer proliferation and metastasis. Cancer Gene Ther. 2023; 30(11): 1530-1542. doi: 10.1038/s41417-023-00657-4.

28. Berquin I M, B Pang, M L Dziubinski, et al. Y-box-binding protein 1 confers EGF independence to human mammary epithelial cells. Oncogene. 2005; 24(19): 3177-86. doi: 10.1038/sj.onc.1208504.

29. Wu J, C Lee, D Yokom, et al. Disruption of the Y-box binding protein-1 results in suppression of the epidermal growth factor receptor and HER-2. Cancer Res. 2006; 66(9): 4872-9. doi: 10.1158/0008-5472.Can-05-3561.

30. Liang C, Y Ma, L Yong, et al. Y-box binding protein-1 promotes tumorigenesis and progression via the epidermal growth factor receptor/AKT pathway in spinal chordoma. Cancer Sci. 2019; 110(1): 166-179. doi: 10.1111/cas.13875.

31. Hussain S A and T Venkatesh YBX1/lncRNA SBF2-AS1 interaction regulates proliferation and tamoxifen sensitivity via PI3K/AKT/MTOR signaling in breast cancer cells. Mol Biol Rep. 2023; 50(4): 3413-3428. doi: 10.1007/s11033-023-08308-5.

32. Prabhu L, R Mundade, B Wang, et al. Critical role of phosphorylation of serine 165 of YBX1 on the activation of NF-κB in colon cancer. Oncotarget. 2015; 6(30): 29396-412. doi: 10.18632/oncotarget.5120.

33. Martin M, L Hua, B Wang, et al. Novel Serine 176 Phosphorylation of YBX1 Activates NF-κB in Colon Cancer. J Biol Chem. 2017; 292(8): 3433-3444. doi: 10.1074/jbc.M116.740258.

34. Hartley A V, B Wang, R Mundade, et al. PRMT5-mediated methylation of YBX1 regulates NF-κB activity in colorectal cancer. Sci Rep. 2020; 10(1): 15934. doi: 10.1038/s41598-020-72942-3.

35. Xu F, M Huang, Q Chen, et al. LncRNA HIF1A-AS1 Promotes Gemcitabine Resistance of Pancreatic Cancer by Enhancing Glycolysis through Modulating the AKT/YB1/HIF1α Pathway. Cancer Res. 2021; 81(22): 5678-5691. doi: 10.1158/0008-5472.Can-21-0281.

36. Ruan H, L Bao, Z Tao, et al. Flightless I Homolog Reverses Enzalutamide Resistance through PD-L1-Mediated Immune Evasion in Prostate Cancer. Cancer Immunol Res. 2021; 9(7): 838-852. doi: 10.1158/2326-6066.Cir-20-0729.

37. Tuerxun T, X Li, F Lou, et al. YBX1 Protects against Apoptosis Induced by Oxygen-Glucose Deprivation/Reoxygenation in PC12 Cells via Activation of the AKT/GSK3β Pathway. Folia Biol (Praha). 2021; 67(4): 150-157.

38. Shi Q, Y He, S He, et al. RP11-296E3.2 acts as an important molecular chaperone for YBX1 and promotes colorectal cancer proliferation and metastasis by activating STAT3. J Transl Med. 2023; 21(1): 418. doi: 10.1186/s12967-023-04267-4.

39. Wang M, M Dai, D Wang, et al. The long noncoding RNA AATBC promotes breast cancer migration and invasion by interacting with YBX1 and activating the YAP1/Hippo signaling pathway. Cancer Lett. 2021; 512: 60-72. doi: 10.1016/j.canlet.2021.04.025.

40. Bendrick J L, L A Eldredge, E I Williams, et al. Desmoplakin Harnesses Rho GTPase and p38 Mitogen-Activated Protein Kinase Signaling to Coordinate Cellular Migration. J Invest Dermatol. 2019; 139(6): 1227-1236. doi: 10.1016/j.jid.2018.11.032.

41. Kam C Y, A D Dubash, E Magistrati, et al. Desmoplakin maintains gap junctions by inhibiting Ras/MAPK and lysosomal degradation of connexin-43. J Cell Biol. 2018; 217(9): 3219-3235. doi: 10.1083/jcb.201710161.

42. Wang Y, C Li, L Shi, et al. Integrin β1D Deficiency-Mediated RyR2 Dysfunction Contributes to Catecholamine-Sensitive Ventricular Tachycardia in Arrhythmogenic Right Ventricular Cardiomyopathy. Circulation. 2020; 141(18): 1477-1493. doi: 10.1161/circulationaha.119.043504.

43. Dubash A D, C Y Kam, B A Aguado, et al. Plakophilin-2 loss promotes TGF-β1/p38 MAPK-dependent fibrotic gene expression in cardiomyocytes. J Cell Biol. 2016; 212(4): 425-38. doi: 10.1083/jcb.201507018.

44. Celeghin R, G Risato, G Beffagna, et al. A novel DSP zebrafish model reveals training- and drug-induced modulation of arrhythmogenic cardiomyopathy phenotypes. Cell Death Discov. 2023; 9(1): 441. doi: 10.1038/s41420-023-01741-2.

45. Miyazaki Y J, J Hamada, M Tada, et al. HOXD3 enhances motility and invasiveness through the TGF-beta-dependent and -independent pathways in A549 cells. Oncogene. 2002; 21(5): 798-808. doi: 10.1038/sj.onc.1205126.

46. Olcum M, S Fan, L Rouhi, et al. Genetic inactivation of β-catenin is salubrious, whereas its activation is deleterious in desmoplakin cardiomyopathy. Cardiovasc Res. 2023; 119(17): 2712-2728. doi: 10.1093/cvr/cvad137.

47. Yang Y, J Fan, H Xu, et al. Long noncoding RNA LYPLAL1-AS1 regulates adipogenic differentiation of human mesenchymal stem cells by targeting desmoplakin and inhibiting the Wnt/β-catenin pathway. Cell Death Discov. 2021; 7(1): 105. doi: 10.1038/s41420-021-00500-5.

48. Tanimura S, J Hashizume, N Arichika, et al. ERK signaling promotes cell motility by inducing the localization of myosin 1E to lamellipodial tips. J Cell Biol. 2016; 214(4): 475-89. doi: 10.1083/jcb.201503123.

49. Zhang Y, F Cao, Y Zhou, et al. Tail domains of myosin-1e regulate phosphatidylinositol signaling and F-actin polymerization at the ventral layer of podosomes. Mol Biol Cell. 2019; 30(5): 622-635. doi: 10.1091/mbc.E18-06-0398.

50. Dean J L, G Sully, A R Clark, et al. The involvement of AU-rich element-binding proteins in p38 mitogen-activated protein kinase pathway-mediated mRNA stabilisation. Cell Signal. 2004; 16(10): 1113-21. doi: 10.1016/j.cellsig.2004.04.006.

51. Li M Z, E J Liu, Q Z Zhou, et al. Intracellular accumulation of tau inhibits autophagosome formation by activating TIA1-amino acid-mTORC1 signaling. Mil Med Res. 2022; 9(1): 38. doi: 10.1186/s40779-022-00396-x.

52. Gottschald O R, V Malec, G Krasteva, et al. TIAR and TIA-1 mRNA-binding proteins co-aggregate under conditions of rapid oxygen decline and extreme hypoxia and suppress the HIF-1α pathway. J Mol Cell Biol. 2010; 2(6): 345-56. doi: 10.1093/jmcb/mjq032.

53. Tang Y, X Zhang, W Ge, et al. Knockdown of LAP2α inhibits osteogenic differentiation of human adipose-derived stem cells by activating NF-κB. Stem Cell Res Ther. 2020; 11(1): 263. doi: 10.1186/s13287-020-01774-9.

54. Dorner D, S Vlcek, N Foeger, et al. Lamina-associated polypeptide 2alpha regulates cell cycle progression and differentiation via the retinoblastoma-E2F pathway. J Cell Biol. 2006; 173(1): 83-93. doi: 10.1083/jcb.200511149.

55. Bao K, Q Zhang, S Liu, et al. LAP2α preserves genome integrity through assisting RPA deposition on damaged chromatin. Genome Biol. 2022; 23(1): 64. doi: 10.1186/s13059-022-02638-6.

56. Fan A H, X Zhao, H Liu, et al. eEF1A1 promotes colorectal cancer progression and predicts poor prognosis of patients. Cancer Med. 2023; 12(1): 513-524. doi: 10.1002/cam4.4848.

57. Wu W, J Xu, D Gao, et al. TOPK promotes the growth of esophageal cancer in vitro and in vivo by enhancing YB1/eEF1A1 signal pathway. Cell Death Dis. 2023; 14(6): 364. doi: 10.1038/s41419-023-05883-0.

58. Su D, R Wang, G Chen, et al. FBXO32 Stimulates Protein Synthesis to Drive Pancreatic Cancer Progression and Metastasis. Cancer Res. 2024; 84(16): 2607-2625. doi: 10.1158/0008-5472.Can-23-3638.

59. Huang J, C Zheng, J Shao, et al. Overexpression of eEF1A1 regulates G1-phase progression to promote HCC proliferation through the STAT1-cyclin D1 pathway. Biochem Biophys Res Commun. 2017; 494(3-4): 542-549. doi: 10.1016/j.bbrc.2017.10.116.

60. Bao Y, T L Zhao, Z Q Zhang, et al. High eukaryotic translation elongation factor 1 alpha 1 expression promotes proliferation and predicts poor prognosis in clear cell renal cell carcinoma. Neoplasma. 2020; 67(1): 78-84. doi: 10.4149/neo_2019_190224N158.

61. Blanch A, F Robinson, I R Watson, et al. Eukaryotic translation elongation factor 1-alpha 1 inhibits p53 and p73 dependent apoptosis and chemotherapy sensitivity. PLoS One. 2013; 8(6): e66436. doi: 10.1371/journal.pone.0066436.

62. Qiao L, N Xie, Y Li, et al. Downregulation of HNRNPM inhibits cell proliferation and migration of hepatocellular carcinoma through MAPK/AKT signaling pathway. Transl Cancer Res. 2022; 11(7): 2135-2144. doi: 10.21037/tcr-21-2484.

63. Zhu G Q, Y Wang, B Wang, et al. Targeting HNRNPM Inhibits Cancer Stemness and Enhances Antitumor Immunity in Wnt-activated Hepatocellular Carcinoma. Cell Mol Gastroenterol Hepatol. 2022; 13(5): 1413-1447. doi: 10.1016/j.jcmgh.2022.02.006.

64. Shou Y, C Yue, Q Wang, et al. circPTPN12 promotes the progression and sunitinib resistance of renal cancer via hnRNPM/IL-6/STAT3 pathway. Cell Death Dis. 2023; 14(3): 232. doi: 10.1038/s41419-023-05717-z.

65. Zhong H, Q Li, S Pei, et al. hnRNPM suppressed IRF7-mediated IFN signaling in the antiviral innate immunity in triploid hybrid fish. Dev Comp Immunol. 2023; 148: 104915. doi: 10.1016/j.dci.2023.104915.

66. Passacantilli I, P Frisone, E De Paola, et al. hnRNPM guides an alternative splicing program in response to inhibition of the PI3K/AKT/mTOR pathway in Ewing sarcoma cells. Nucleic Acids Res. 2017; 45(21): 12270-12284. doi: 10.1093/nar/gkx831.

67. He Z, Y Zhong, P Regmi, et al. Exosomal long non-coding RNA TRPM2-AS promotes angiogenesis in gallbladder cancer through interacting with PABPC1 to activate NOTCH1 signaling pathway. Mol Cancer. 2024; 23(1): 65. doi: 10.1186/s12943-024-01979-z.

68. Li J, M Pei, W Xiao, et al. The HOXD9-mediated PAXIP1-AS1 regulates gastric cancer progression through PABPC1/PAK1 modulation. Cell Death Dis. 2023; 14(5): 341. doi: 10.1038/s41419-023-05862-5.

69. Zhu C, C Wang, X Wang, et al. PABPC1 silencing inhibits pancreatic cancer cell proliferation and EMT, and induces apoptosis via PI3K/AKT pathway. Cytotechnology. 2024; 76(3): 351-361. doi: 10.1007/s10616-024-00626-1.

70. Zhang H, H B Xu, E Kurban, et al. LncRNA SNHG14 promotes hepatocellular carcinoma progression via H3K27 acetylation activated PABPC1 by PTEN signaling. Cell Death Dis. 2020; 11(8): 646. doi: 10.1038/s41419-020-02808-z.

71. Lee S Y, H M Jeon, M K Ju, et al. Wnt/Snail signaling regulates cytochrome C oxidase and glucose metabolism. Cancer Res. 2012; 72(14): 3607-17. doi: 10.1158/0008-5472.Can-12-0006.

72. Arreola A, C L Cowey, J L Coloff, et al. HIF1α and HIF2α exert distinct nutrient preferences in renal cells. PLoS One. 2014; 9(5): e98705. doi: 10.1371/journal.pone.0098705.

73. Yang Y, B Wang, H Dong, et al. The mitochondrial enzyme pyruvate carboxylase restricts pancreatic β-cell senescence by blocking p53 activation. Proc Natl Acad Sci U S A. 2024; 121(44): e2401218121. doi: 10.1073/pnas.2401218121.

74. Lao-On U, P Rojvirat, P Chansongkrow, et al. c-Myc directly targets an over-expression of pyruvate carboxylase in highly invasive breast cancer. Biochim Biophys Acta Mol Basis Dis. 2020; 1866(3): 165656. doi: 10.1016/j.bbadis.2019.165656.

75. Chen F, S Wang, C Zeng, et al. Silencing circSERPINE2 restrains mesenchymal stem cell senescence via the YBX3/PCNA/p21 axis. Cell Mol Life Sci. 2023; 80(11): 325. doi: 10.1007/s00018-023-04975-6.

76. Xie J, H Zhang, K Wang, et al. M6A-mediated-upregulation of lncRNA BLACAT3 promotes bladder cancer angiogenesis and hematogenous metastasis through YBX3 nuclear shuttling and enhancing NCF2 transcription. Oncogene. 2023; 42(40): 2956-2970. doi: 10.1038/s41388-023-02814-3.

77. Liu X, J Meng, X Liao, et al. A de novo missense mutation in MPP2 confers an increased risk of Vogt-Koyanagi-Harada disease as shown by trio-based whole-exome sequencing. Cell Mol Immunol. 2023; 20(11): 1379-1392. doi: 10.1038/s41423-023-01088-9.

78. Miao D, Q Wang, J Shi, et al. N6-methyladenosine-modified DBT alleviates lipid accumulation and inhibits tumor progression in clear cell renal cell carcinoma through the ANXA2/YAP axis-regulated Hippo pathway. Cancer Commun (Lond). 2023; 43(4): 480-502. doi: 10.1002/cac2.12413.

79. Shalhout S Z, P Y Yang, E M Grzelak, et al. YAP-dependent proliferation by a small molecule targeting annexin A2. Nat Chem Biol. 2021; 17(7): 767-775. doi: 10.1038/s41589-021-00755-0.

80. Yan X, D Zhang, W Wu, et al. Mesenchymal Stem Cells Promote Hepatocarcinogenesis via lncRNA-MUF Interaction with ANXA2 and miR-34a. Cancer Res. 2017; 77(23): 6704-6716. doi: 10.1158/0008-5472.can-17-1915.

81. Duan H, L Chen, L Qu, et al. Mycoplasma hyorhinis infection promotes NF-κB-dependent migration of gastric cancer cells. Cancer Res. 2014; 74(20): 5782-94. doi: 10.1158/0008-5472.can-14-0650.
