# Supplementary figures and images for: A novel lncRNA, Lnc21q22.11, suppresses gastric cancer growth by inhibiting MEK/ERK pathway
Source: Epigenetics. 2025 Jun 2;20(1):2512764. doi: 10.1080/15592294.2025.2512764 (PMC12140449; doi:10.1080/15592294.2025.2512764)

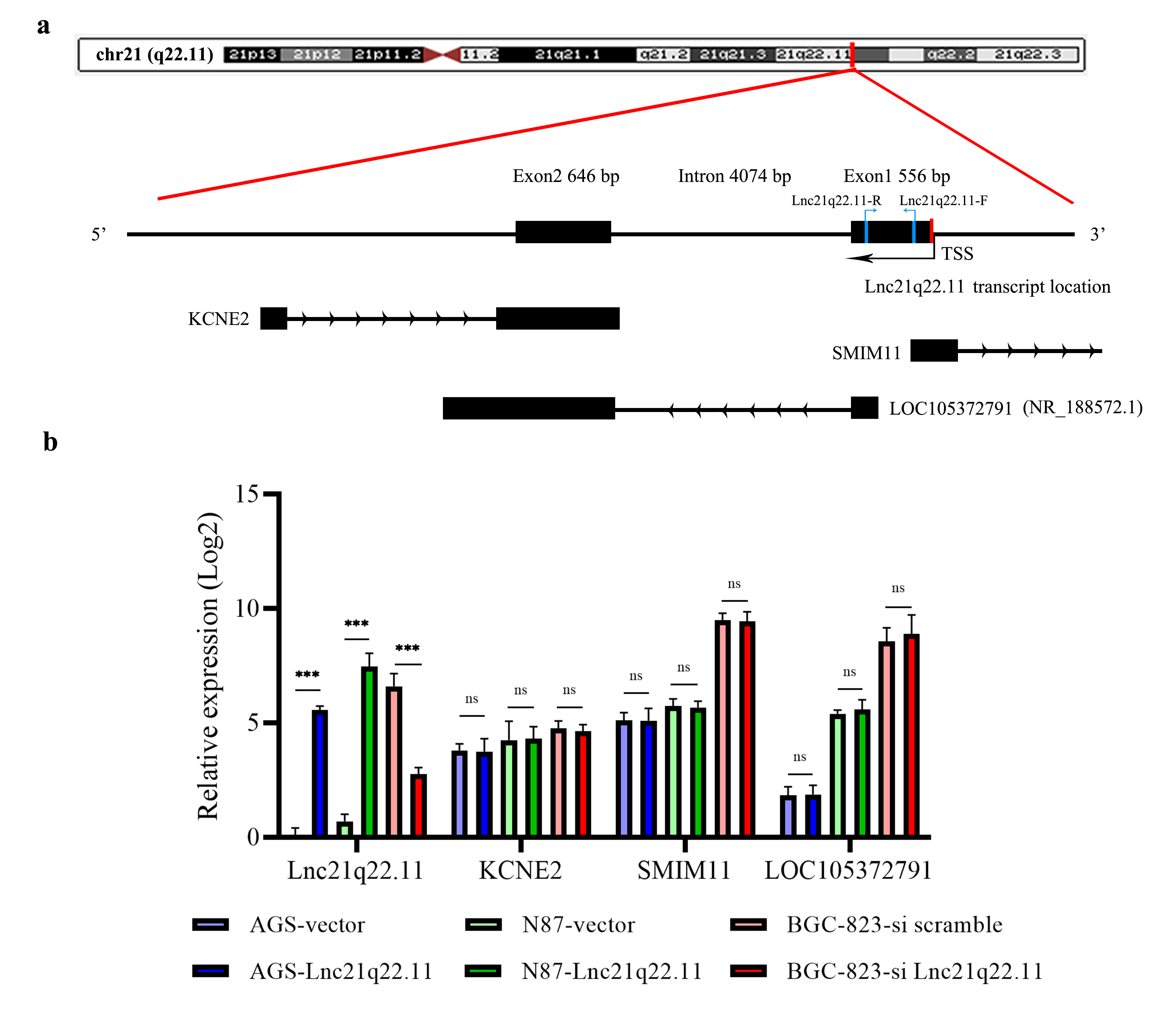

Supplement: Supplemental Material [file KEPI_A_2512764_SM2130.zip › Supplementary files/Supplementary_figure_1.tif]

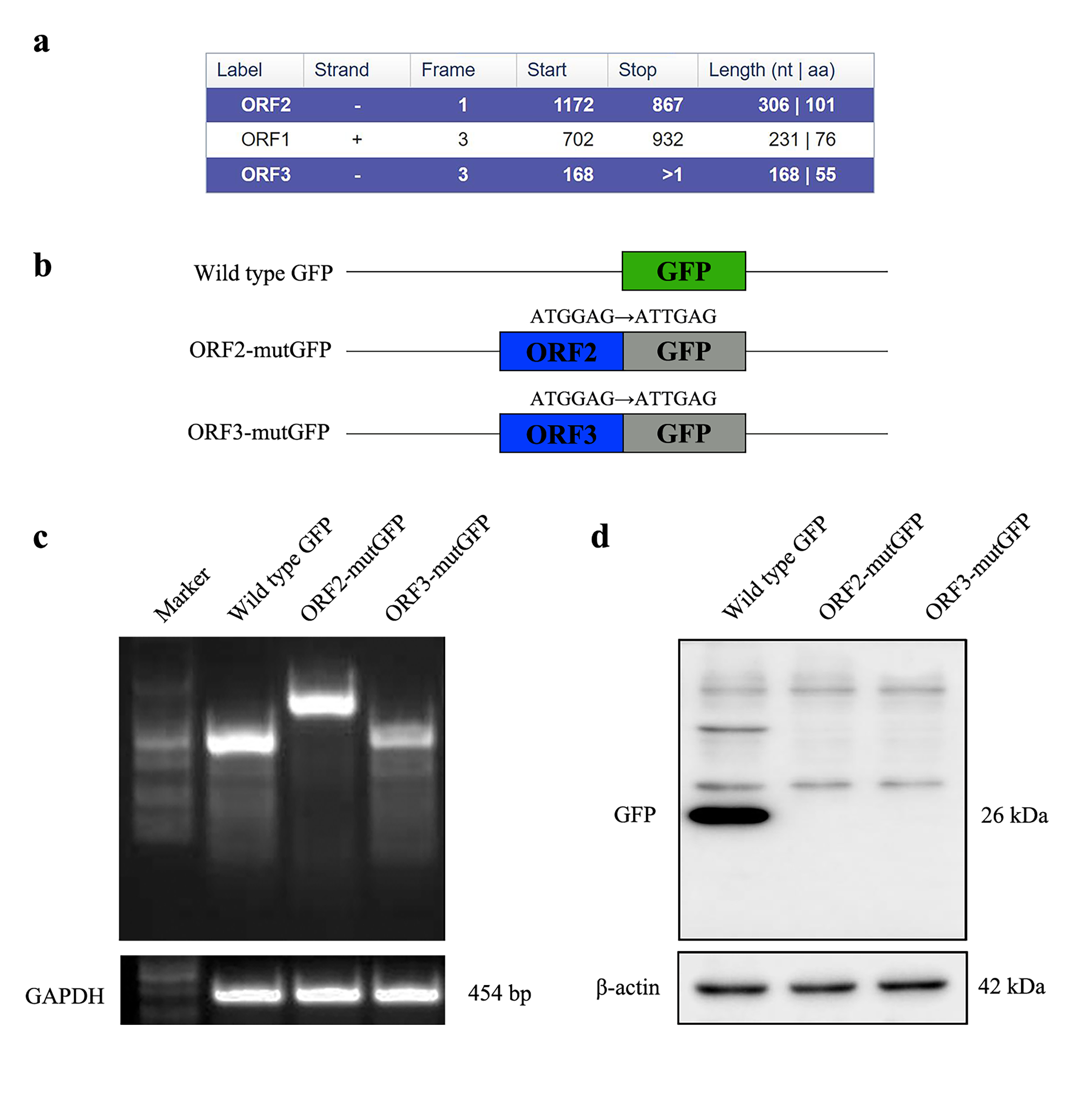

Supplement: Supplemental Material [file KEPI_A_2512764_SM2130.zip › Supplementary files/Supplementary_figure_2.tif]

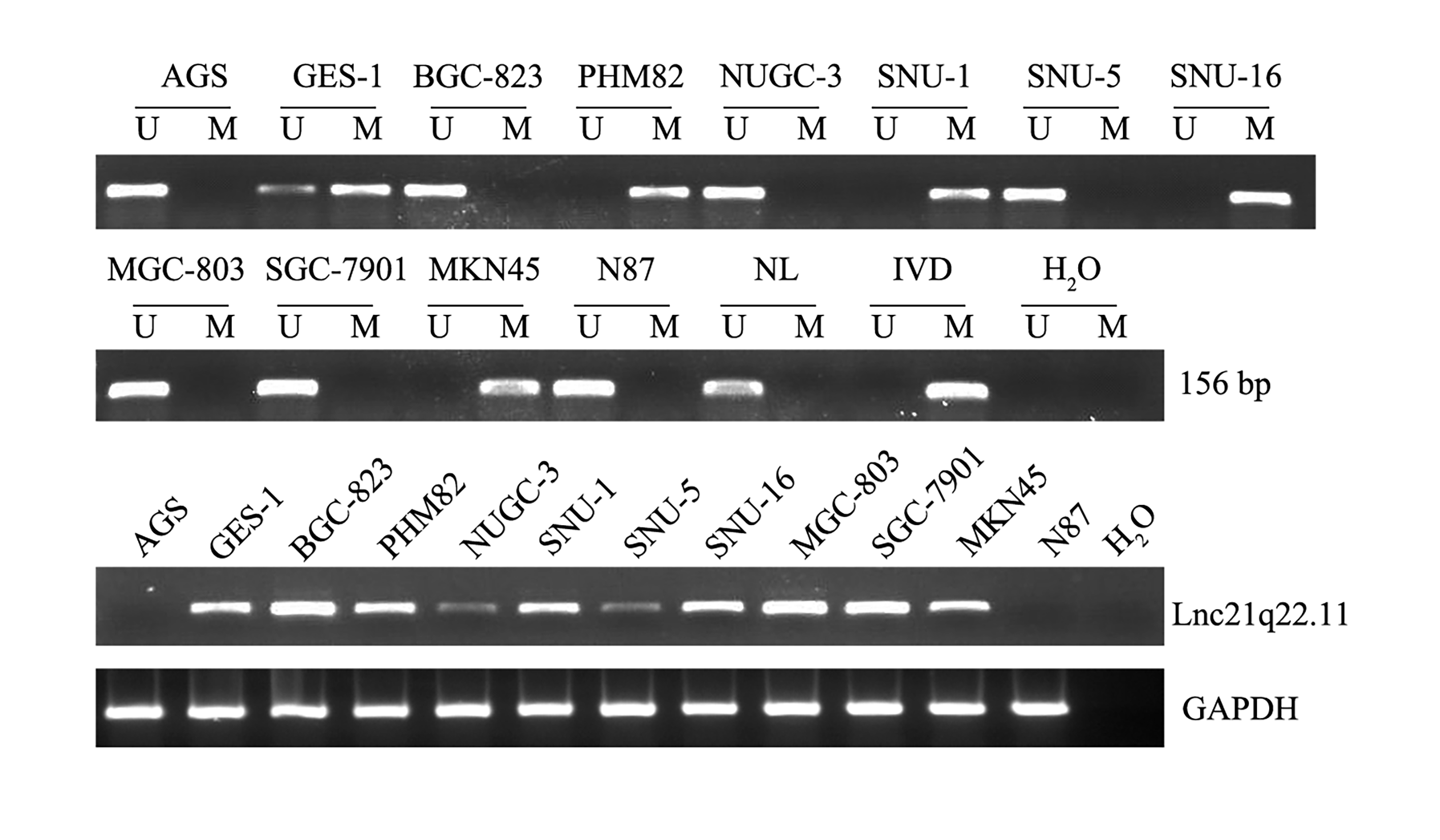

Supplement: Supplemental Material [file KEPI_A_2512764_SM2130.zip › Supplementary files/Supplementary_figure_3.tif]

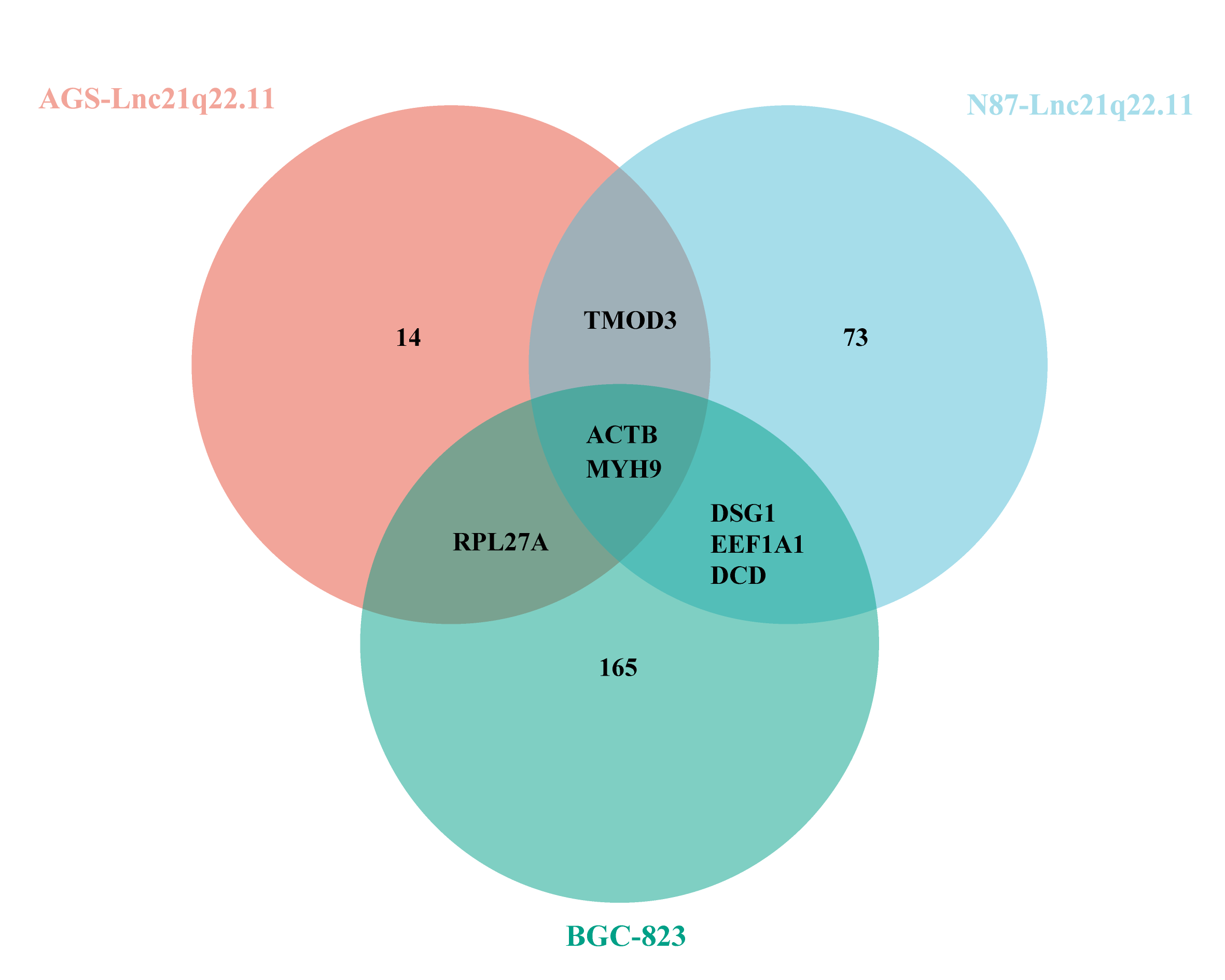

Supplement: Supplemental Material [file KEPI_A_2512764_SM2130.zip › Supplementary files/Supplementary_figure_4.tif]

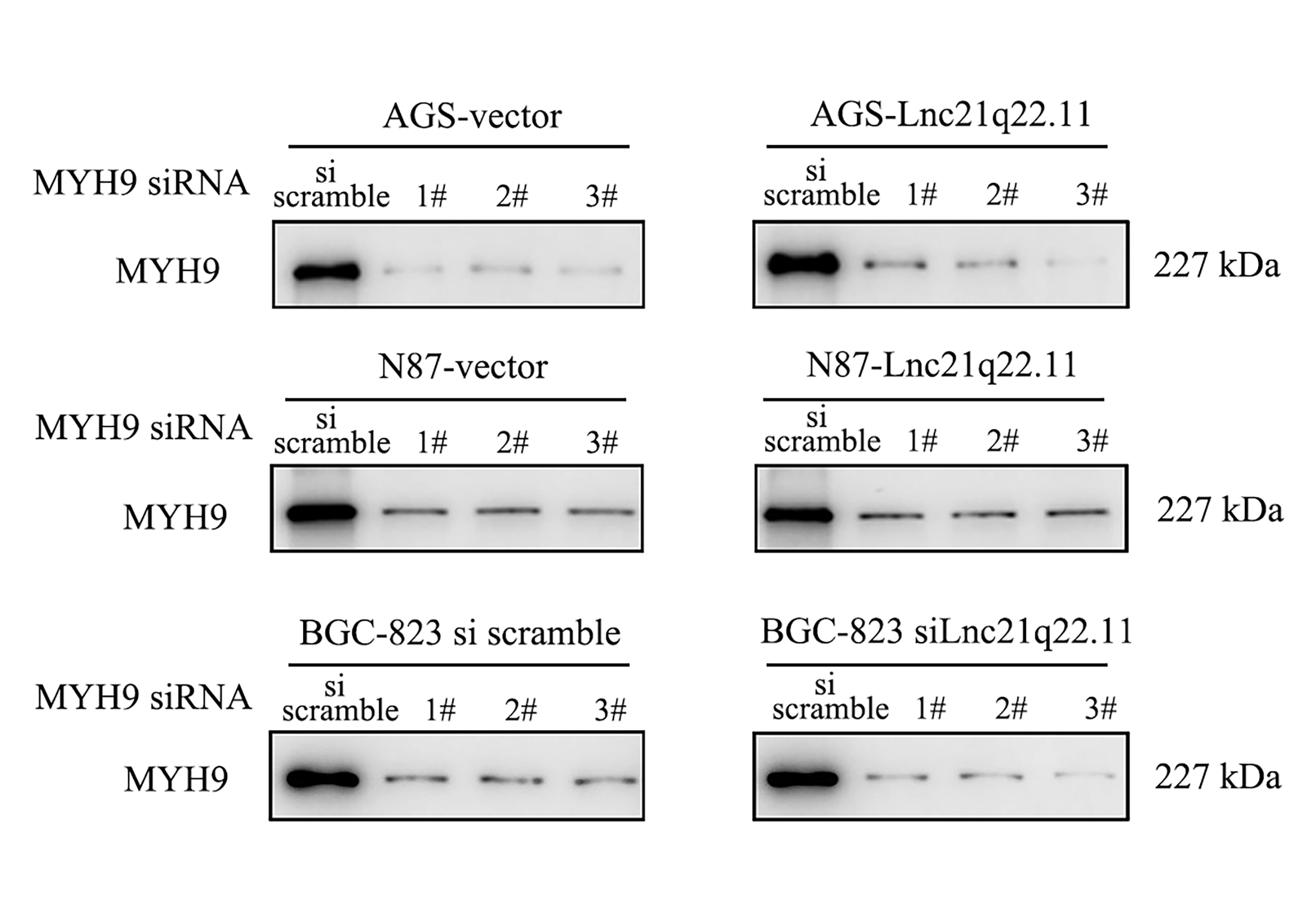

Supplement: Supplemental Material [file KEPI_A_2512764_SM2130.zip › Supplementary files/Supplementary_figure_5.tif]
